# Enhanced Hole Mobility of p-Type Materials by Molecular Engineering for Efficient Perovskite Solar Cells

**DOI:** 10.1021/acsomega.3c04088

**Published:** 2023-07-20

**Authors:** Tamer Yeşil, Adem Mutlu, Sirin Siyahjani Gültekin, Zeynep Gülay Günel, Ceylan Zafer

**Affiliations:** Solar Energy Institute, Ege University, Izmir 35100, Turkey

## Abstract

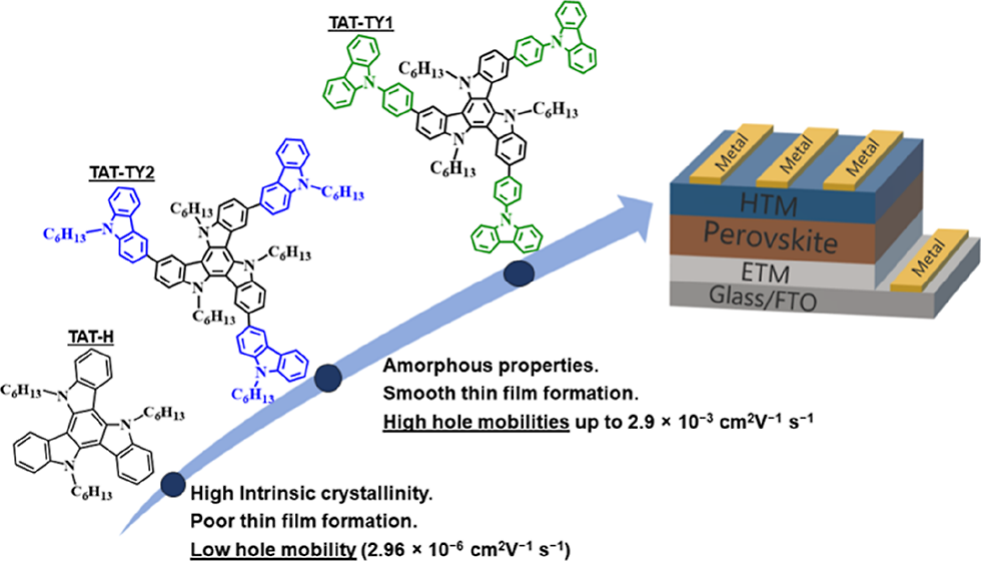

Star-shaped triazatruxene derivative hole-transporting materials (HTMs), namely, 3,8,13-tris(4-(8a,9a-dihydro-9*H*-carbazol-9-yl)phenyl)-5,10,15-trihexyl-10,15-dihydro-5*H*-diindolo[3,2-a:3′,2′-c]carbazole (TAT-TY1) and 3,8,13-tris(4-(8a,9a-dihydro-9*H*-carbazol-9-yl)phenyl)-5,10,15-trihexyl-10,15-dihydro-5*H*-diindolo[3,2-a:3′,2′-c]carbazole (TAT-TY2), containing electron-rich triazatruxene cores and donor carbazole moieties, were synthesized and successfully used in triple-cation perovskite solar cells. All the HTMs were obtained from relatively inexpensive precursor materials using well-known synthesis procedures and uncomplicated purification steps. All the HTMs, including the 5,10,15-trihexyl-10,15-dihydro-5H-diindolo[3,2-a:3’,2’-c]carbazole (TAT-H) main core, had suitable highest occupied molecular orbitals (HOMOs) for perovskite (TAT-H: −5.15 eV, TAT-TY1: −5.17 eV, and TAT-TY2: −5.2 eV). Steady-state and time-resolved photoluminescence results revealed that hole transport from the valence band of the perovskite into the HOMO of the new triazatruxene derivatives was more efficient than with TAT-H. Furthermore, the substitution of *n*-hexylcarbazole and 9-phenylcarbazole in triazatruxene altered the crystalline nature of the main core, resulting in a smooth and pinhole-free thin-film morphology. As a result, the hole mobilities of TAT-TY1 and TAT-TY2 were measured to be one order of magnitude higher than that of TAT-H. Finally, TAT-TY1 and TAT-TY2 achieved power conversion efficiencies of up to 17.5 and 16.3%, respectively, compared to the reference Spiro-OMeTAD. These results demonstrate that the new star-shaped triazatruxene derivative HTMs can be synthesized without using complicated synthesis strategies by controlling the intrinsic morphology of the TAT-H main core.

## 1 Introduction

In the last decade, consumption of fossil fuel resources and environmental pollution have motivated scientific studies to focus on renewable energy sources. Solar energy gained popularity due to its widespread availability, sustainability, and long-term carbon-free nature. The approach of converting electromagnetic radiation into electrical energy is the most promising long-term approach.^1^ In 1991, Grätzel and O’Regan demonstrated that efficiencies of up to 7.2%^2,3^ could be achieved using solid hole-transporting materials (HTMs), instead of liquid electrolytes in the dye-sensitized solar cells. Since Miyasaka et al. first used perovskite materials in solar energy applications in 2009, perovskite solar cells (PSCs) have become the most intensively studied research topic in their field owing to their unique optical and electrical properties such as high charge density (ε: 10–5), adjustable energy levels, and low exciton binding energy (E < 0.005 eV) 4. Thus, efficiencies ranging from 3.8 to 25.2% were achieved in a short time.^4,5^

The HTMs used in the architecture of high-efficiency PSCs perform three main tasks. These tasks include transporting photo-induced holes in the perovskite layer to the back metal electrode, blocking the transport of electrons to the back electrode, and preventing direct contact between the perovskite layer and the metal electrode.^7^ To fulfill these tasks, the highest occupied molecular orbital (HOMO) and lowest unoccupied molecular orbital (LUMO) energy levels of HTMs should align with the energy levels of perovskites to prevent electrons from reaching the back electrode and facilitate effective hole extraction. Furthermore, they should exhibit good solubility in organic solvents such as chlorobenzene (CB), chloroform (CF), and dichloromethane (DCM) to enable the formation of smooth thin films, minimizing surface traps and preventing contact between the perovskite layer and the metal electrode. Lastly, HTMs should possess high intrinsic hole mobility and conductivity.^8^ To achieve these desired properties, the energy level of the selected central molecule should be compatible with that of the perovskite to enable efficient hole transfer between the perovskite layer and the HTM.^9^ Furthermore, they should be highly soluble in organic solvents to obtain an ideal thin film. Currently, the most widely used organic HTMs for PSCs are 2,2′,7,7′-tetrakis[*N*,*N*-di(4-methoxyphenyl)amino]-9,9′-spirobifluorene (Spiro-OMeTAD) and poly[bis(4-phenyl)(2,5,6-trimethylphenyl)amine] (PTAA). However, these organic molecules are relatively expensive. Additionally, the Spiro-OMeTAD molecule does not have the desired hole mobility and electrical conductivity unless doping is performed (10^–5^ cm^2^ V^–1^ s^–1^; 10^–7^ S cm^–1^).^6^ Considering all these features, the 10,15-dihydro-5*H*-diindolo[3,2-a:3′,2′-c] carbazole (triazatruxene) molecule has become an important building block in this field.

Triazatruxene derivative molecules with three different molecular topologies have been synthesized and applied in the field of organic electronics in the literature. In 2015, Rakstys et al. synthesized a series of star-shaped HTMs by centering the triazatruxene molecule and extending the conjugation with donor methoxybenzene groups at carbon atoms 3, 8, and 13. By utilizing the energy level alignment and anchoring effect of electron-rich methoxy groups attached to the aromatic benzene ring,^10,11^ they achieved an efficiency of up to 18.3% and demonstrated the potential of triazatruxene-derived star-shaped HTMs for PSCs.^9^ In 2018, Connel et al. utilized relatively inexpensive precursor materials to synthesize star-shaped triazatruxene derivatives consisting of three hexyl side chains on indole nitrogen and three terminal *tert*-butoxy styrene groups. This approach eliminated the need for column chromatography during the purification process of the final product and then successfully achieved a conversion efficiency of up to 20.3%.^12^ Thereafter, Illicachi et al. employed dumbbell-shaped triazatruxene derivatives composed of triazatruxene units to highlight the significance of electron-donating π linkers between the triazatruxene molecules.^13^ Finally, Kil et al. synthesized a series of dopant-free HTMs and investigated their photovoltaic performance compared to doped and undoped Spiro-OMeTAD.^14^

According to the literature, the triazatruxene main core possesses readily available high hole mobility,^15^ HOMO energy level, electrochemically stable heterocyclic nitrogen atoms, and a planar extended π-system with an electron-rich structure. However, its high intrinsic crystalline nature results in challenges in obtaining smooth and pinhole-free thin films.^7^ In this study, star-shaped and π-extended triazatruxene derivatives, namely, TAT-TY1 and TAT-TY2, were synthesized using the Suzuki–Miyaura cross-coupling reaction. Their electrochemical, photophysical, and morphological properties were analyzed, and the TAT-H main core was used as a reference for understanding the structure–performance relationship. Apart from the discussed synthesis strategies, we further extended the π-conjugation of the triazatruxene main core by incorporating electron-rich and cost-effective phenyl carbazole and *n*-hexyl carbazole donor moieties. This strategy allowed us to achieve suitable HOMO and LUMO energy levels for the perovskite layer without the need for methoxy and electron-withdrawing acceptor moieties. Furthermore, the high intrinsic crystalline nature of the main core was altered by introducing carbazole moieties, resulting in amorphous, highly smooth, and pinhole-free thin films. This modification was achieved without sacrificing the readily available properties offered by triazatruxene, such as energy levels, electrochemical stability, and hole mobility. As a result, high hole mobilities were measured as 2.9 × 10^–3^ and 1.0 × 10^–3^ cm^2^ V^–1^ s^–1^ for TAT-TY1 and TAT-TY2, respectively. To the best of our knowledge, these hole mobilities are the highest among previously reported triazatruxene derivatives. Finally, the synthesized HTMs were successfully used in triple-cation PSCss, achieving power conversion efficiencies (PCEs) of up to 17.5 and 16.3% for TAT-TY1 and TAT-TY2, respectively.

## 2 Materials and Methods

NaH (60% dispersion in mineral oil), 1-bromohexane (%98), *N*-bromosuccinimide (NBS) *ReagentPlus* (99%), carbazole (≥95%), *n*-buthyllithium solution (2.5 M in hexane), potassium carbonate, sodium hydroxide, trimethyl borate (≥98%), and phosphorous(V) oxychloride (99.999%) were purchased from MERC. Hexane, suitable for high-performance liquid chromatography (HPLC), ≥97.0%, dichloromethane, suitable for HPLC ≥99.8%; *N*,*N*-dimethylformamide, anhydrous, 99.8%; chloroform, suitable for HPLC, ≥99.8%; and tetrahydrofuran, ≥99.0% were purchased from MERCK. Spiro-OMeTAD was purchased from Borun. Bis(trifluoromethane)sulfonimide lithium salt (Li-TFSI, 99.0%) was purchased from Acros. Lead(II) bromide (PbBr_2_, 99.999%), methylammonium bromide (MABr, >99.5%), and formamidinium iodide (FAI, >99.5%) were bought from Lumtec. Lead iodide (PbI_2_, 99.99%) was bought from TCI. Titanium isopropoxide (Ti[OCH(CH3)_2_]_4_, 97%), hydrochloric acid (HCl, 37%), 2-propanol (For HPLC, 99.9%), chlorobenzene (anhydrous 99.8%), acetonitrile (99.8%), 4-tertbutylpyridine (99.0%), and cesium iodide (CsI, 99.999%) were purchased from Sigma-Aldrich. Fluorine-doped tin oxide (FTO) glass slides (2.5 × 2.5 cm^2^, FTO OPV-FTO22-15, sheet resistance of 14 Ω sq^–1^) were bought from OPVTech.

### 2.1 Synthesis and Characterization

**Figure 1:**
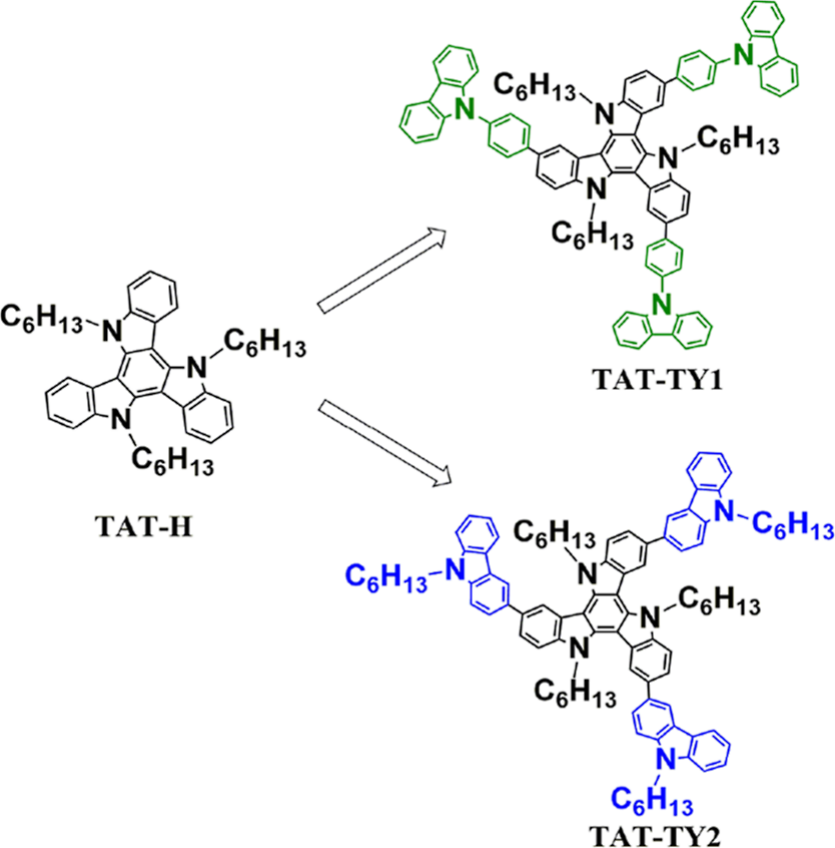
Chemical structures of TAT-H and the synthesized star-shaped HTMs.

The triazatruxene main core (1) was synthesized via a one-pot intermolecular trimerization reaction by refluxing 2-oxindole in phosphoryl chloride (POCl_3_). The resulting intermediate product was subsequently *n*-hexylated under inert conditions to make it soluble in organic solvents, such as CB. It is also known that n-hexyl chains protect perovskite layers from moisture. Thereafter, *n*-hexylated triazatruxene (2) was brominated using NBS to obtain a brominated product (3), which was prepared for the Suzuki–Miyaura cross-coupling reaction to yield TAT-TY1 and TAT-TY2. Subsequently, *n*-hexylcarbazole-3-ylboronic acid and 4-(9*H*-carbazol-9-yl) phenylboronic acid were synthesized from carbazole and 9-(4-bromophenyl) carbazole using well-known synthesis steps. Finally, TAT-TY1 and TAT-TY2 were synthesized using the triple Suzuki–Miyaura cross-coupling reaction (Figure 1). The synthesis methods are discussed in detail in Figures S1–S5. The chemical structures of all the intermediate products were characterized using 1H NMR and 13C NMR spectroscopy and were found to be identical to the previous results reported in the literature (Figures S6–S23).^9,12−14,16−19^ Additionally, liquid chromatography–electrospray ionization–quadrupole time-of-flight–mass spectrometry (LC–ESI–QTOF–MS) analysis was conducted on TAT-TY1 and TAT-TY2 (Figures S24–S31).

### 2.2 Electrochemical and Photophysical Characterization

Absorption spectra were recorded for HTMs at a concentration of 1 × 10^–5^ M at 25 °C in anhydrous CHCl_3_. Measurements were performed using a Perkin Elmer Lambda 950 UV–vis spectrophotometer over a wavelength range of 250–800 nm. Cyclic voltammetry (CV) measurements were recorded for HTMs at a concentration of 1 × 10^–5^ M at 25 °C in argon-flushed anhydrous CHCl_3_ using 0.1 M TBAPF_6_ as a supporting electrolyte. A triple-electrode system consisting of a glassy carbon working electrode, an Ag wire pseudo-reference electrode, and a platinum wire counter electrode was used. Photoluminescence (PL) and time-resolved photoluminescence (TRPL) measurements of the thin films were conducted using monochromatic light as an optical excitation source with a 450 W Xe arc lamp.

### 2.3 Morphology and Thermal Characterization

X-ray diffraction (XRD) measurements were carried out using a Rigaku XRD Ultima IV diffractometer. The surface topology of the thin films and root mean square (rms) values were obtained using a Park Systems NX20 atomic force microscope with a PPP-NCHR 5M probe. Scanning electron microscopy (SEM) images were captured using a Thermo Scientific Apreo S instrument. A Perkin Elmer Pyris 6 differential scanning calorimeter was used for the calorimetric characterization of HTMs. Differential scanning calorimetry (DSC) curves of all HTMs were recorded at a heating rate of 10 °C/min under a nitrogen flow. The weights of the samples were maintained in the range of 3–4 mg. Thermogravimetric analysis (TGA) was performed using a TGA Q500 device. The measurements were taken under a nitrogen flow, and the samples were maintained in the range of 3–4 mg. The hydrophobicity of the synthesized HTMs was investigated using a Krüss DSA255 contact angle measurement device by coating them on top of the perovskite layer. The thin-film resistivity of the HTMs was measured using the four-probe method by coating the HTMs on top of the insulating glass, and the corresponding conductivities were calculated.

## 3 Results and Discussion

### 3.1 Optical Properties

**Figure 2:**
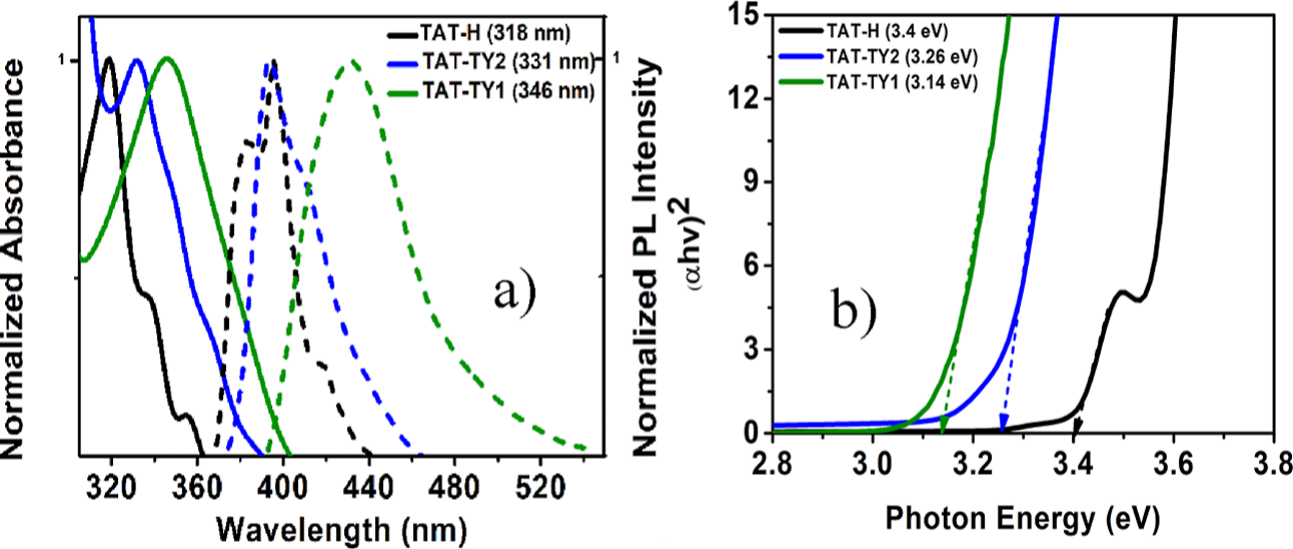
(a) Normalized absorption (solid lines), (b) PL spectra (dashed lines), and Tauc plot of TAT-H and the synthesized HTMs with carbazole moieties in 1 × 10^–5^ M CF.

Spectrophotometric measurements were conducted to measure the optical band gap and energy-level transitions of the synthesized molecules. Characteristic π–π* energy-level transitions of the triazatruxene core were observed in the range of 318–346 nm (Figure 2a).^20^ Furthermore, the π–π* energy transitions of the carbazole moieties were also observed at 292 and 300 nm (Figure S32).^21^ As expected, the molecular absorption shifted to the visible region due to the extension of conjugation from carbon atoms 3, 8, and 13 using n-hexyl carbazole and 9-phenylcarbazole moieties. According to solution-phase PL spectroscopy measurements, Stokes shifts of 67 and 87 nm were observed for the TAT-TY2 and TAT-TY1 molecules, respectively. These results suggest that the 9-phenylcarbazole and n-hexyl carbazole moieties undergo rotational changes in their geometry under ultraviolet light excitation. The redshift in the absorption of the materials into the visible region indicates a significant decrease in the optical band gap corresponding to the HTMs. Therefore, the Tauc plot method was used to calculate the optical band gaps.^22^ The optical band gaps of the materials were calculated as 3.4, 3.26, and 3.14 eV for TAT-H, TAT-TY1, and TAT-TY2, respectively, by using the tangent drawn to the absorption curve at the longest wavelength (Figure 2b).

### 3.2 Experimental Energy Levels

**Figure 3:**
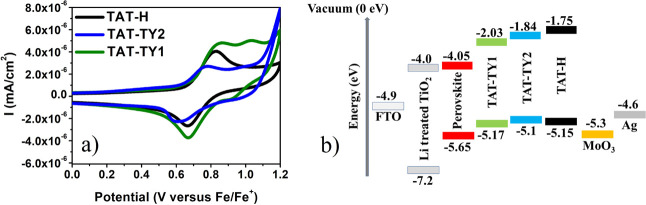
(a) Cyclic voltammograms and (b) energy-level alignment diagram of TAT-H and the HTMs in relation to fluorine-doped tin oxide (FTO), titanium dioxide (TiO_2_), perovskite, and MoO_3_/Ag metal contact.

The HOMO energy levels of the synthesized molecules were determined using CV technique.^23^ The measurements were conducted using a platinum wire/glassy carbon/silver wire triple-electrode system. Furthermore, a 0.1 M tetrabutylammonium hexafluorophosphate (TBAPF_6_) solution was employed as the supporting electrolyte in CF. The HOMO energy levels of the synthesized materials were derived from the ground-state oxidation potentials estimated by CV, with a ferrocene/ferrocenium redox couple serving as the reference (Figure 3a). It was observed that the HOMO energy levels of the TAT-TY1 and TAT-TY2 molecules did not exhibit significant changes compared to that of the TAT-H reference molecule. This result indicates that the HOMO energy levels were primarily influenced by the central triazatruxene core. The redox peaks of the TAT-H and TAT-TY1 demonstrated reversibility, indicating the electrochemical stability of the synthesized HTMs. The experimentally calculated HOMO energy levels of TAT-H, TAT-TY1, and TAT-TY2 were found to be compatible with those of the triple-cation perovskite layer, which consists of Cs_0.05_(_FA0.83_MA_0.17_)_0.95_Pb(I_0.83_Br_0.17_)_3_, facilitating efficient photogenerated hole transfer at the perovskite/HTM interface (Figure 3b). The energy levels and optical bandgaps of the HTMs are summarized in Table 1.

**Table 1:** Summary of the Energy Levels Estimated from Cyclic Voltammograms and the Optical Band Gaps of the Synthesized HTMs

ID	*E*_ox_ (V)	*E*_ox 1/2_	*E*_HOMO_ (-eV)	*E*_LUMO_ (-eV)	*E*_g_^opt^ (-eV)
TAT-H	0.67, 0.83	0.75	–5.15	–1.75	–3.4
TAT-TY2	0.62, 0.78	0.7	–5.1	–1.84	–3.26
TAT-TY1	0.67, 0.86	0.77	–5.17	–2.03	–3.14

### 3.3 Steady-State and Time-Resolved PL Properties

**Figure 4:**
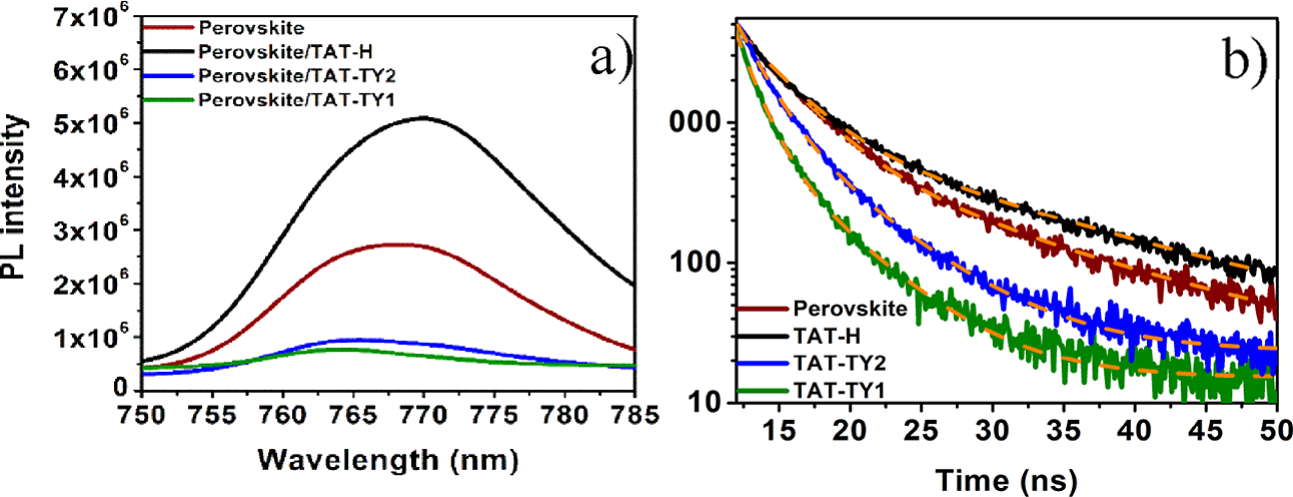
(a) Steady-state PL and (b) TRPL spectra of perovskite, perovskite/TAT-H, perovskite/TAT-TY1, and perovskite/TAT-TY2 films.

Steady-state and time-resolved PL measurements were conducted to investigate the charge transfer properties between the synthesized molecules and the perovskite layer. A triple-cation perovskite film was coated on a glass slide using the spin-coating method and used as a reference in the measurements. The radiative emission signal corresponding to the triple-cation perovskite was observed at 770 nm, which corresponds to its band gap of 1.61 eV (Figure 4a). This emission can also be attributed to radiative recombination. Upon depositing the TAT-TY2 and TAT-TY1 thin films on top of the perovskite layer, a significant quenching in PL intensity was observed. The reduction in radiative PL emissions observed with TAT-TY2 and TAT-TY1 can be attributed to the effective extraction of holes from the HOMO energy level of the perovskite thin film to that of TAT-TY2 and TAT-TY1.^24^ Time-resolved PL measurements were conducted to better understand the recombination kinetics. The resulting emission lifetimes were fitted to a two-component exponential decay model, as shown in Figure 4b and summarized in Table 2. According to the general rate equation of recombination, the value τ_1_ refers to monomolecular charge carrier recombination, which can be exciton-mediated or trap-mediated in perovskite thin films.^25^ On the other hand, the value τ_2_ can be described as bimolecular electron–hole recombination, which is radiative and directly proportional to the electron and hole densities on the perovskite thin film.^26^

**Table 2:** Summary of the τ_1_, τ_2_, and *X*^2^ Values Extracted from the Time-Resolved PL Spectra of the Bare Perovskite and the Perovskite Containing the HTMs (Figure 4)

lifetime	τ_1_ (ns)	τ_2_ (ns)	*X*^2^
perovskite	3.35	12.69	1.117
perovskite + TAT-H	3.20	14.08	1.2897
perovskite + TAT-TY2	1.86	5.46	1.090
perovskite + TAT-TY1	1.37	4.70	1.231

**Table 3:** Summarized Data for *N*_t_ of the Spiro-OMeTAD, TAT-TY1- and TAT-TY2-Based Devices

ID	perovskite thickness (nm)	*N*_t_ (cm^–3^)
TAT-TY1	479	4.33 × 10^16^
TAT-TY2	468	5.01 × 10^16^

The τ_2_ value of the bare perovskite thin film was measured as 12.69 ns. After depositing the HTMs on top of the perovskite thin film, the τ_2_ values were reduced to 5.46 and 4.70 ns for TAT-TY2 and TAT-TY1, respectively. These results revealed that the hole transfer between the perovskite and the TAT-TY2 and TAT-TY1 molecules occurred effectively.^9^ On the other hand, it was observed that the emission intensity increased when the TAT-H molecule was coated compared to that of the reference perovskite thin film. This result indicates that the charge transfer between the perovskite and TAT-H molecule did not occur efficiently, as evidenced by the increase in the τ_2_ value to 14.08 ns compared to the reference.

### 3.4 Thin-Film Structures, Morphologies, and Hole-Transporting Mobilities

**Figure 5:**
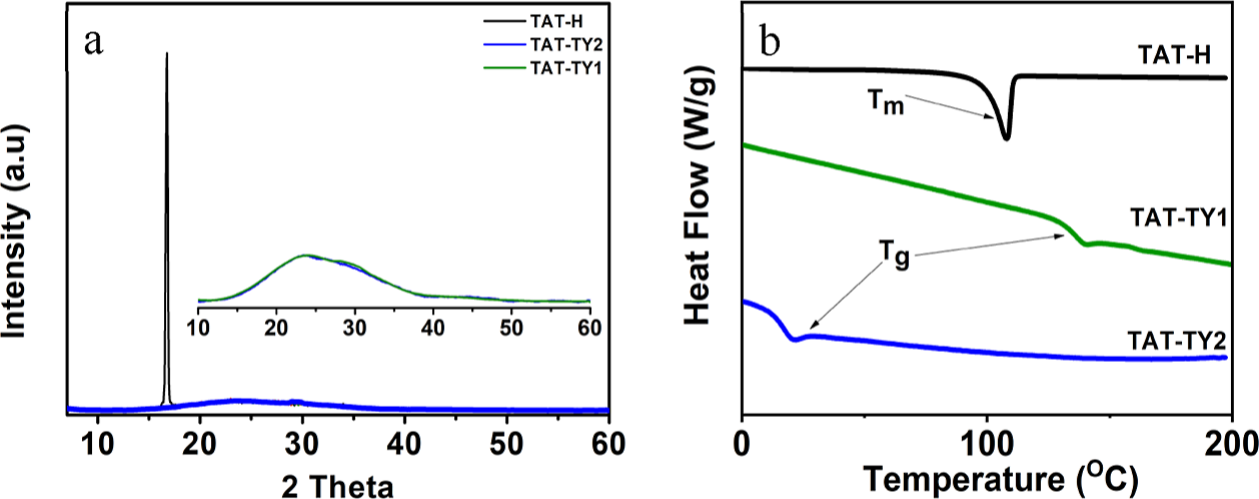
Thin-film XRD (a) and powder DSC measurements of the corresponding HTMs (b). Both measurements revealed that TAT-H had an intrinsic crystallinity, while the synthesized HTMs had relatively amorphous properties.

TAT-H, TAT-TY2, and TAT-TY1 molecules prepared at a 73 mM concentration were coated on a glass slide at 4000 rpm for 40 s, and XRD measurements were recorded to analyze the morphology and structure of the HTMs. The strong signal observed in the thin film coated with the TAT-H molecule indicated that the material had a high crystallinity in the thin-film phase. However, an amorphous glass signal was observed between 20 and 30° from the TAT-TY2- and TAT-TY1-coated glass substrates (Figure 5a). These results indicated that TAT-TY1 and TAT-TY2 molecules have amorphous properties in a thin-film phase. HTMs should exhibit amorphous properties in order to achieve adequate uniformity and surface coverage in PSCs. Furthermore, it is preferable for HTM materials in PSCs to be amorphous to ensure a compatible interface with the perovskite structure, facilitating more efficient charge transfer.^13^ The introduction of phenylcarbazole and n-hexyl carbazole side groups at carbon atoms 3, 8, and 13 led to a transformation in the highly crystalline structure of the TAT-H molecule as the molecular weight increased. Additionally, the presence of long alkyl chains in the TAT-TY2 and TAT-TY1 molecules may have disrupted the crystal structure, resulting in an amorphous thin-film morphology.

The DSC instrument was used for the calorimetric characterization of the triazatruxene-based HTM materials to determine their physical properties, such as glass transition and/or melting point. The DSC curves of all HTMs were recorded at a heating rate of 10 °C/min under a nitrogen flow (Figure 5b). The sample weights were maintained in the range of 3–4 mg. A sharp endothermic peak corresponding to the melting point of TAT-H was observed at 108.06 °C on the DSC curve. This peak indicated the presence of a relatively small and rigid structure, resulting in a crystalline thin-film structure. However, the TAT-TY1 and TAT-TY2 molecules exhibited glass transition temperatures (*T*_g_) of 139.66 and 20.70 °C, respectively, without any melting point (*T*_m_) or crystallization temperature (*T*_c_), indicating their relatively amorphous nature compared to TAT-H. Furthermore, the presence of long alkyl chains on TAT-TY2 decreased molecular interactions through a steric effect, leading to increased disorder and a lower glass transition temperature compared to TAT-TY1.^27^ It is worth noting that the DSC measurements were conducted using powder samples, while the XRD spectra were recorded in the thin-film phase. Both experiments confirmed the relatively amorphous properties of the HTMs in both the thin film phase and powder form, in contrast to the TAT-H main core. TGA of the synthesized HTMs was performed to observe the thermal behavior of the materials. According to the TGA results, both TAT-TY1 and TAT-TY2 exhibited thermal stability up to 428.4 and 322.82 °C, respectively (Figure S34).

**Figure 6:**
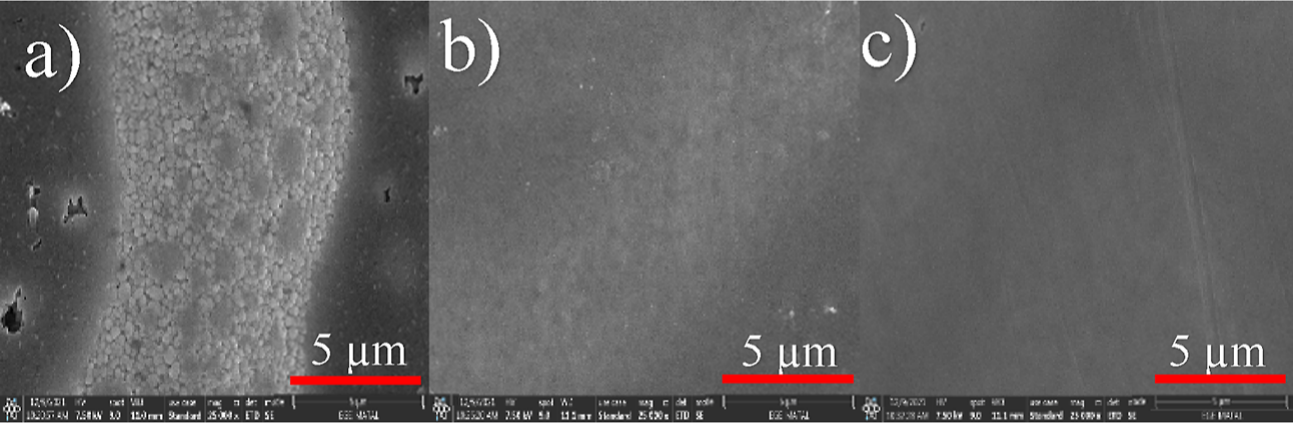
Top-view SEM images of (a) TAT-H, (b) TAT-TY1, and (c) TAT-TY2 on top of the perovskite layer.

The rms values of the synthesized molecules were determined from the atomic force microscopy (AFM) images. The triple-cation perovskite thin film exhibited an rms value of 16.7 nm (Figure S35a). When TAT-H was spin-coated on top of the perovskite layer, the rms value increased to 58.8 nm, and crystal grains and pinholes corresponding to the TAT-H thin film on the perovskite layer were observed (Figure S36b). This increase in surface roughness suggested the presence of direct contact between the perovskite grains and the metal. However, after coating the perovskite surfaces with TAT-TY2 and TAT-TY1, the rms values decreased to 2.9 and 6.2 nm, respectively, indicating a more homogeneous and smoother surface (Figure S36a–d). Furthermore, when spiro-OMeTAD was coated on the perovskite, the rms value was measured as 14.1 nm (Figure S35e). SEM images were also taken. The SEM images revealed poor surface coverage and pinholes on the thin films coated with the TAT-H molecule (Figure 6a). This result can be attributed to the highly crystalline nature of TAT-H. In contrast, homogeneous and good surface coverages were observed on the thin films coated with the TAT-TY2 and TAT-TY1 molecules (Figure 6b,c).

**Figure 7:**
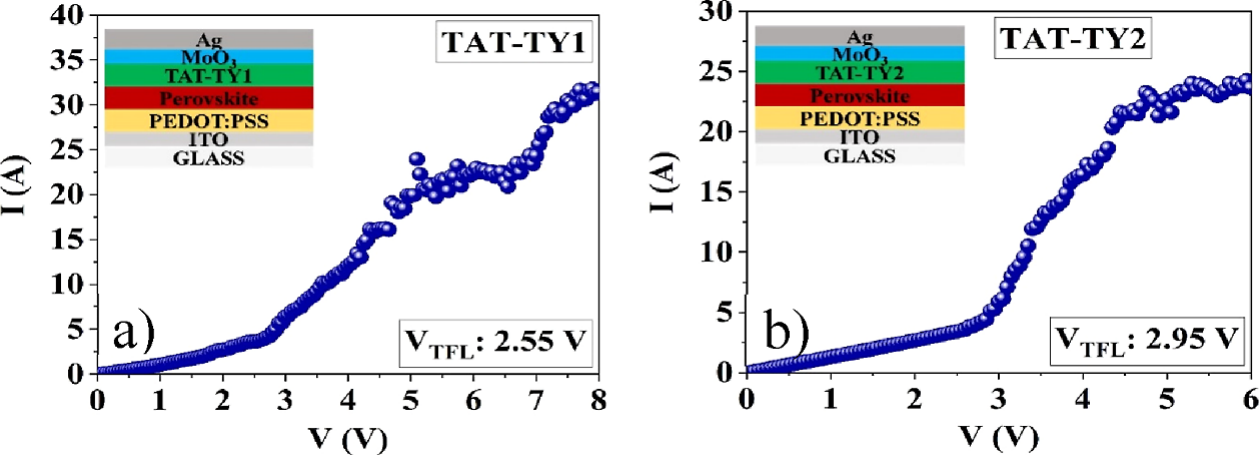
SCLC measurements of the hole-only devices corresponding to (a) TAT-TY1 and (b) TAT-TY2 (the inset showing the device structures).

Hole-only devices with the ITO/PEDOT/PSS/perovskite/HTM/MoO_3_/Ag configuration were fabricated to investigate the state of charge traps for the HTM molecules. *J*–*V* plots (Figure 7) were measured in a glovebox under dark conditions. The trap density can be calculated using the following equation:^28^
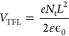
1where *N*_t_ is the trap state density, *L* is the perovskite film thickness, *e* represents the fundamental charge, ε is the dielectric constant of the active layer, and ε_0_ is the permittivity of free space, respectively. Additionally, the dielectric constant for the triple cation perovskite was assumed to be 32.5,^29^ and the thicknesses of the perovskite thin films were measured as 468 and 479 nm using a profilometer. The *V*_TFL_ values of the devices fabricated with TAT-TY1 and TAT-TY2 were measured as 2.55 and 2.95 V, respectively. According to space charge-limited current (SCLC) theory, the trap densities corresponding to the devices fabricated with TAT-TY1 and TAT-TY2 were calculated as 4.33 × 10^16^ and 5.01 × 10^16^ cm^–3^, respectively (Table 3). Lower PL intensity, lower lifetime, and lower *N*_t_ values were observed for TAT-TY1 compared to that for TAT-TY2. These results indicate that better charge transfer occurs at the perovskite/TAT-TY1 interface than with TAT-TY2. Additionally, the *N*_t_ value was calculated as 4.59 × 10^16^ cm^–3^ (*V*_TFL_ = 2.7 V) for Spiro-OMeTAD (Figure S33). Therefore, perovskite devices prepared with TAT-TY1 demonstrate lower hole injection than Spiro-OMeTAD.

**Figure 8:**
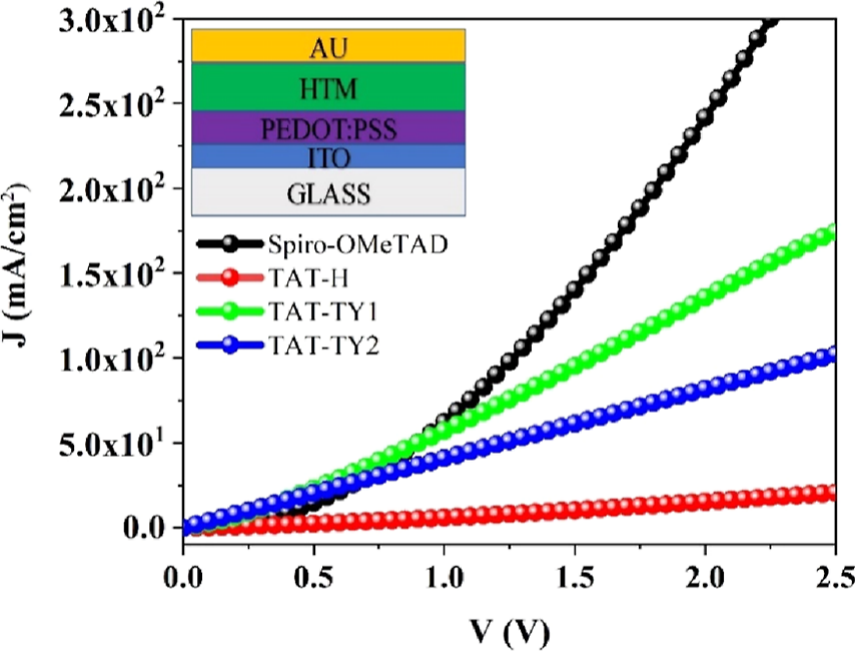
Characteristic *J*–*V* measurements of the hole-only devices fabricated with TAT-H and the synthesized HTMs.

The hole transport mobilities of doped and undoped HTMs were measured using SCLC measurements with an indium-doped tin oxide ITO/PEDOT/PSS/HTM/Au device architecture.^30,31^ The *J*–*V* characteristics of the hole-only devices were fitted using the Mott–Gurney law, expressed as
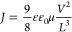
2where ε is the dielectric constant of the active layer, μ is the mobility, ε_0_ is the permittivity of free space, and *L* is the thickness of the active layer (Figure 8). All measurements were performed in the dark, and the voltage was scanned from 0 to 5 V. The thicknesses of all the films were measured using a profilometer to calculate the hole mobilities. The hole mobilities of TAT-H, TAT-TY1, and TAT-TY2 were measured as 4.0 × 10^–6^, 2.9 × 10^–3^, and 1.0 × 10^–3^ cm^2^ V^–1^ s^–1^, respectively. Due to their effective hole extraction properties and adequate thin-film-forming abilities, the TAT-TY1 and TAT-TY2 molecules exhibited hole transport mobilities that were 10^3^ times higher than that of the TAT-H molecule, similar to doped Spiro-OMeTAD (7.2 × 10^–3^ cm^2^ V^–1^ s^–1^).^32^

### 3.5 Hydrophobicity and Thin-Film Conductivity Measurements

The static contact angles of doped HTMs on top of the perovskite layer were measured as 95, 90, 94, and 80° for TAT-H, Spiro-OMeTAD, TAT-TY1, and TAT-TY2, respectively. Furthermore, the contact angles of undoped HTMs were measured as 90, 82, 79.5, and 83°, respectively (Figure S37). All HTMs exhibited hydrophobic behavior due to the presence of long alkyl chains on the −N positions of triazatruxene and carbazole moieties. Consequently, we found that doped HTMs were effective in preventing the permeation of moisture into the perovskite lattice.

The bulk resistivities of the doped HTMs were measured as 1598, 292,965, 7135, and 6835 Ωm for Spiro-OMeTAD, TAT-H, TAT-TY1, and TAT-TY2, respectively. On the other hand, the undoped thin-film resistivities were measured as 23,290, 452,950, 602,305, and 523,505 Ωm, respectively. As expected, the thin-film resistivities of the HTMs decreased upon doping with Li-TFSI, which can be similarly explained by the controlled oxidation of Spiro-OMeTAD triggered by doping in the solution phase.^33^ Consequently, the conductivities of the HTMs increased by approximately one order of magnitude. The hole mobilities also increased proportionally and were in good agreement with the conductivity results (Table 4).

**Table 4:** Summary of Hole Mobility, Resistivity, and Thin-Film Thicknesses of HTMs

doped HTM’s	hole mobility (cm^2^ V^–1^ s^–1^)	conductivity (S/cm)	thin-film thickness (nm)
Spiro-OMeTAD	7.2 × 10^–3^	6.2 × 10^–4^	115
TAT-H	4.0 × 10–^6^	3.4 × 10^–6^	102
TAT-TY1	2.9 × 10–^3^	1.4 × 10^–5^	119
TAT-TY2	1.0 × 10–^3^	1.5 × 10^–5^	122

undoped HTMs	hole mobility (cm^2^ V^–1^ s^–1^)	conductivity (S/cm)	thin film thickness (nm)
Spiro-OMeTAD	1.78 × 10^–5^	4.2 × 10^–5^	109
TAT-H	2.96 × 10–6	2.2 × 10^–6^	115
TAT-TY1	1.08 × 10–5	1.6 × 10^–6^	128
TAT-TY2	2.24 × 10–5	1.9 × 10^–6^	130

### 3.6 Photovoltaic Device Characteristics

**Figure 9:**
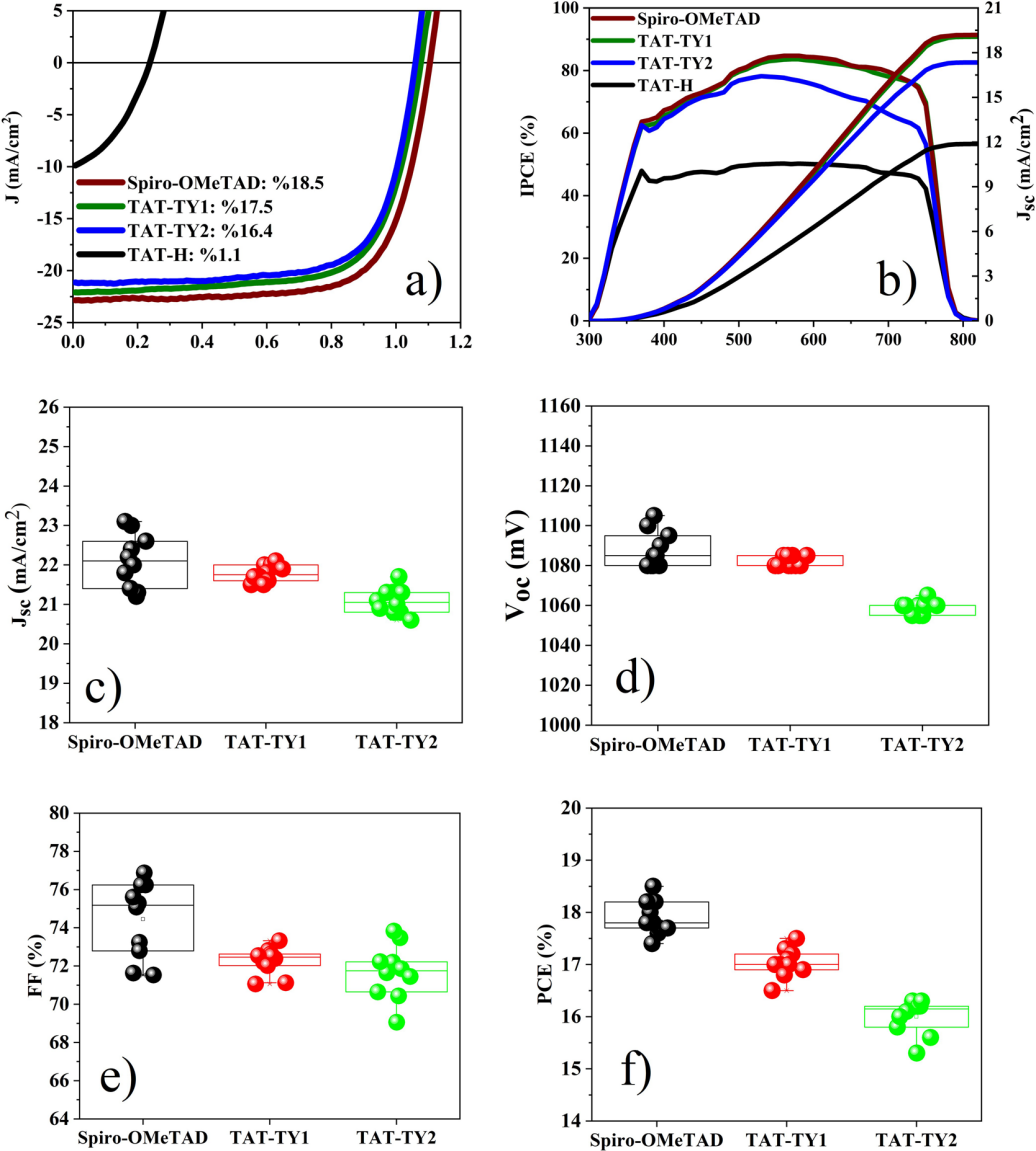
(a) Reverse bias *J*–*V* characteristics of PSCs containing the doped HTMs and (b) the corresponding integrated *J*_sc_ values obtained from the IPCE measurements. Device performance statistics (10 devices) for PSCs prepared with Spiro-OMeTAD, TAT-TY1, and TAT-TY2: (c) *J*_sc_, (d) *V*_oc_, (e) FF, and (f) PCE.

Solar cells were fabricated using the FTO/Li-treated c-TiO_2_/perovskite/HTM (TAT-H, TAT-TY1, and TAT-TY2)/MoO_3_/Ag architecture to evaluate the photovoltaic performance of the new doped and undoped HTMs. The detailed device fabrication and results for the undoped HTMs are provided in the Supporting Information (Table S2). All *J*–*V* measurements were conducted with a voltage step of 0.05 V (Figure 9a), and the photovoltaic parameters of the PSCs are summarized in Table 5.

**Table 5:** *J*–*V* Characteristics of the Best-Performing PSCs Containing the Doped HTMs under Forward and Reverse Bias Conditions

molecules	scan direction	*J*_sc_ (mA/cm^2^)	*V*_oc_ (mV)	FF (%)	best PCE	HI
Spiro-OMeTAD	forward	23.0	1090	62.3	15.7	
	reverse	23.0	1105	72.8	18.5	0.0135
TAT-H	forward	9.7	220	44.5	0.95	
	reverse	10.1	245	26.7	1.10	0.1376
TAT-TY1	forward	22.0	1080	65.2	15.5	
	reverse	22.1	1080	73.3	17.5	0.0045
TAT-TY2	forward	21.3	1040	68.7	15.2	
	reverse	21.3	1060	72.2	16.3	0.0188

The devices fabricated with TAT-TY1 and TAT-TY2 exhibited lower open-circuit voltage (*V*_oc_) values (∼20 and ∼40 mV, respectively) compared to the control device. These differences could be attributed to the lower-lying HOMO level of Spiro-OMeTAD (∼5.22 eV) compared to both the synthesized HTMs. The thin-film thicknesses of TAT-TY1 and TAT-TY2 were measured as 120 and 220 nm, respectively. The fill factor (FF) values of TAT-TY1 and TAT-TY2 were slightly higher than that of the control device under forward and reverse bias conditions. This difference can be attributed to the relatively greater film thickness of the control device. As the thin-film thickness increases, the charge recombination resistance gradually decreases, resulting in a slight decrease in the FF value.^34^ Both TAT-TY1 and TAT-TY2 molecules had similar HOMO energy levels, rms values, τ_2_ values, and thin-film qualities in the SEM images, with slight differences. Furthermore, the hole transport mobility of TAT-TY1 was found to be slightly higher than that of TAT-TY2 due to the lower film thickness of TAT-TY1 compared to that of TAT-TY2. According to the DSC results, TAT-TY2 had a lower glass transition temperature (20.70 °C) than those of TAT-TY1 and the control device, which may have negatively affected the performance of the device under forward and reverse bias conditions. Apart from the synthesized HTMs, we observed a disordered thin-film morphology of TAT-H with pinholes, which may result in local short-circuits due to direct contact between the perovskite grains and the top electrode. Consistent with the DSC and XRD results, the intrinsic crystallinity of the molecule resulted in a poorer thin-film formation compared to the other HTMs.^35^

According to the calculated hysteresis values summarized in Table 5 and in Figure S38, the solar cell produced with TAT-TY2 exhibited the least hysteresis. The incident photon-to-current conversion efficiency (IPCE) spectra obtained in the range of 300–820 nm and the integrated current density (*J*_sc_) values are shown in Figure 9b. The *J*_sc_ values obtained from the IPCE measurements were 19.6, 12.03, 19.35, and 17.6 mA/cm^2^ for the control device, TAT-H, TAT-TY1, and TAT-TY2, respectively. The measurement *J*_sc_ errors were also calculated as 11.3, 19.1, 15.89, and 17.37% for the control device, TAT-H, TAT-TY1, and TAT-TY2, respectively.^29,36,37^

The deviations of the IPCE curves between the integrated *J*_sc_ values and the *J*–*V* measurements were calculated in the range of 12–21%, and these values are comparable to the errors reported in previous literature. Theoretically, these deviations can be mainly attributed to the following three reasons: (1) the approximately 10–15% loss in IPCE is due to reflection and absorption by the FTO glass. (2) Full irradiation applied in the *J*–*V* test produces loaded charge carriers and provides more efficient charge transport/collection than wavelength-dependent light in IPCE measurement. (3) Full irradiation includes near-infrared light, which generates heat in the IPCE measurement, and an increase in temperature leads to an increase in *J*_sc_.^29,38^ Additionally, the device performance statistics of 10 devices for PSCs prepared with Spiro-OMeTAD, TAT-TY1, and TAT-TY2 are shown in Figure 9c–f.

### 3.7 Cost Analysis of the Materials

In Table S1, a cost analysis of TAT-TY1 and TAT-TY2 molecules, including all chemicals, was performed using previously reported cost analysis models.^12,39,40^ The cost of the materials, including the final products, was calculated and reported as $/g and $/mL based on Sigma-Aldrich base prices. According to the cost analysis results, the product cost of triazatruxene was calculated as $109.4/g, which was the highest among all synthetic steps, mainly due to the increasing cost of the 2-oxindole starting material ($8/g). On the other hand, the cost of the reagents used in synthesizing the carbazole moieties was calculated as $4.1/g, $2.89/g, and $18.57/g for *n*-hexylcarbazole, 3-bromo-*n*-hexylcarbazole, and *n*-hexylcarbazole-3-ylboronic acid, respectively. Due to the relatively low synthesis cost of carbazole moieties, the final product cost of TAT-TY1 and TAT-TY2 was calculated as $273.12/g and $269.6/g, respectively, which is on average $346.64/g cheaper than the commercial price of Spiro-OMeTAD ($618.00/g). The cost of the final products could be further reduced by carefully optimizing the isolated reaction efficiencies and recycling the dichloromethane used in the purification of triazatruxene, and the other solvents used in column chromatography.

## 4 Conclusions

In summary, we synthesized two new star-shaped triazatruxene derivative HTMs and characterized their electrochemical and photophysical properties. We found that the HOMO energy levels of the new HTMs, dominated by a central triazatruxene core, were suitable for effective hole extraction from triple-cation perovskite layers. Time-resolved and steady-state PL measurements demonstrated efficient hole injection from the perovskite’s HOMO level to that of the HTMs. Additionally, we observed a redshift in the ultraviolet–visible spectrum due to the increased conjugation length. Morphological studies revealed that introducing n-hexyl-substituted carbazole and phenyl carbazole moieties at the 3rd, 8th, and 13th positions of the main core altered the intrinsic crystalline morphology of the TAT-H molecule, resulting in a smooth and pinhole-free thin-film formation. Consequently, high hole mobilities (2.9 × 10^–3^, 1 × 10^–3^ cm^2^ V^–1^ s^–1^) were obtained from SCLC measurements for TAT-TY1 and TAT-TY2, respectively. Finally, the photovoltaic performance of the PSCs fabricated with TAT-TY1 and TAT-TY2 was evaluated, yielding efficiencies of up to 17.5 and 16.4%, respectively, compared to those fabricated with Spiro-OMeTAD. Our study demonstrates that by utilizing the readily available intrinsic electrochemical and photophysical properties of TAT-H molecules and modifying their highly crystalline nature to a relatively amorphous form using donor n-hexyl carbazole and phenyl carbazole moieties, it is possible to synthesize new star-shaped triazatruxene derivative HTMs for highly efficient PSCs without the need for complex synthesis strategies or expensive precursors.

## Supporting Information Available

NMR spectroscopy, HR-MS, absorption spectroscopy, SCLC measurement of Spiro-OMeTAD, TGA results of HTMs, AFM, SEM images of TAT-H (10, 20, and 100 μm), contact angle measurements of doped and undoped HTMs, cost analysis of products, and hysteresis curves of HTMs (PDF)
