# Supplementary material for: Enhanced Hole Mobility of p-Type Materials by Molecular Engineering for Efficient Perovskite Solar Cells
Source: ACS Omega. 2023 Jul 20;8(30):27784–93. doi: 10.1021/acsomega.3c04088 (PMC10399180; doi:10.1021/acsomega.3c04088)
Supplement: Supplementary file 1 — ao3c04088_si_001.pdf [file ao3c04088_si_001.pdf]

Supporting Information

Enhanced Hole Mobility of p-Type Materials by Molecular Engineering for Efficient Perovskite Solar Cells

Tamer YEŞİL[1], Adem MUTLU[1], Sirin Siyahjani GÜLTEKİN[1], Zeynep Gülay GÜNEL[1], Ceylan ZAFER[1*]

[1]*Solar Energy Institute, Ege University, 35100, Izmir, Turkey*

Corresponding authors

C. Zafer. E-mail: ceylan.zafer@ege.edu.tr

Experimental Section

Synthesis and characterization of intermediate and final molecules were carried out by using previously described methods [1–8] and were olso explained in detail as following:

Synthesis of 3,8,13-tribromo-5,10,15-trihexyl-10,15-dihydro-5H-diindolo[3,2-a:3',2'-c]carbazole (3):

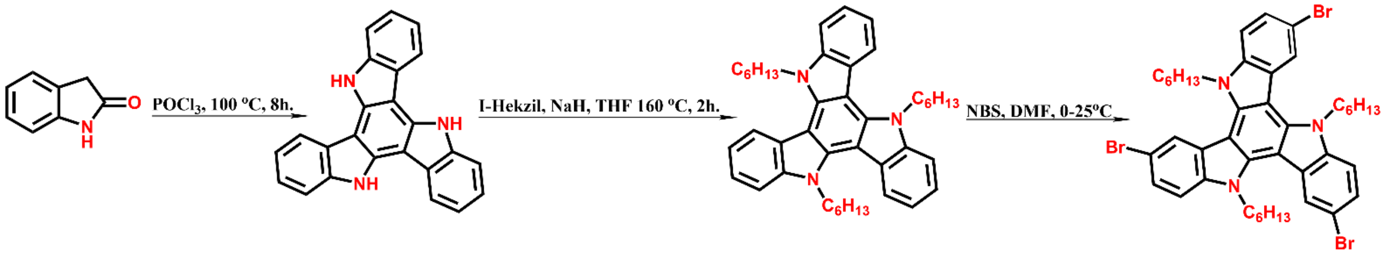

Fig. S1. Synthesis scheme of 3,8,13-tribromo-5,10,15-trihexyl-10,15-dihydro-5H-diindolo[3,2-a:3',2'-c]carbazole.

Synthesis of 10,15-dihydro-5H-diindolo[3,2-a:3',2'-c]carbazole (1): Oxindole (10 g; 75 mol) was dissolved in $POCl_3$ (50 mL), and the mixture was stirred for 8 h at 100 °C. After the reaction was completed, the reaction mixture was cooled to room temperature. Thereafter, the reaction mixture was poured into ice water and neutralized to pH = 7–8 using a saturated KOH solution. After neutralization, the precipitate was filtered to yield a brown solid crude product. The brown solid was dissolved with MeOH and absorbed into silica gel, and the solvent was evaporated. The crude product was purified using flash chromatography with DCM as an eluent. An off-white solid was obtained. Yield: 40% ^{1}H NMR (DMSO-d_6), δ (ppm): 11.85(s, 3H), 8.68(d, 3H), 7.73(d, 3H), and 7.40–7.30(m, 6H).

TAT-H (2): 10,15-dihydro-5H-diindolo[3,2-a:3',2'-c]carbazole(350 mg; 1 mmol) was suspended in DMF(10 mL) in a two-neck flame-dried round bottom flask. NaH (71 mg; 3.5 mmol) was added at room temperature and stirred for 30 min. After the reagents were dissolved, 1-bromohexane (4 mmol, 660 mg, and 0.560 mL) was added slowly. The mixture was refluxed for 2 h at 160 °C under a nitrogen atmosphere. The reaction was cooled to room temperature and then added to water. An aqueous phase was extracted using DCM and dried over $MgSO_4$. The solvent was removed under reduced pressure, and the residue was subsequently

purified using column chromatography (Hexane: DCM [4:1]) to yield a white solid product. (500 mg, 75%) ^{1}H NMR ($CDCl_3$) δ (ppm): 8.30(d, 3H), 7.64(d, 3H), 7.45(t, 3H), 7.35(t, 3H), 4.92(m, 6H), 2.01(p, 6H), 1.30–1.22(m, 18H), and 0.81(t, 9H); ^{13}C NMR: 141.05, 138.93, 123.51, 122.71, 121.53, 119.65, 110.53, 47.07, 31.50, 29.85, 26.41, 22.51, and 14.00.

Synthesis of 3,8,13-tribromo-5,10,15-trihexyl-10,15-dihydro-5H-diindolo[3,2-a:3',2'-c]carbazole (3): TAT-H (500 mg, 0.84 mol, and 1 equiv) was dissolved in 40 mL of $CHCl_3$ in a 100 mL round bottom flask, and an NBS solution (462 mg; 2.6 mol; 3.1 equiv) in DMF (5 mL) was added dropwise to the mixture at 0 °C. Thereafter, the reaction mixture was stirred at room temperature for 1 h. The reaction was poured into water, extracted with DCM (30 mL ×3), and the organic phase was dried with $MgSO_4$. The crude product was purified using silica gel column chromatography, with hexane/DCM (9:1) as an eluent, yielding a pale yellow solid product. (675 mg, %80) ^{1}H NMR ($CDCl_3$) δ (ppm): 7.93(d, 3H), 7.64(s, 3H), 7.39(d, 3H), 4.60(m, 6H), 1.84(m, 6H), 1.26–1.17(m, 18H), and 0.80(t, 9H); ^{13}C NMR: 141.53, 138.29, 122.63, 122.31, 121.61, 116.44, 113.19, 102.55, 46.72, 31.31, 29.69, 22.49, and 13.94.

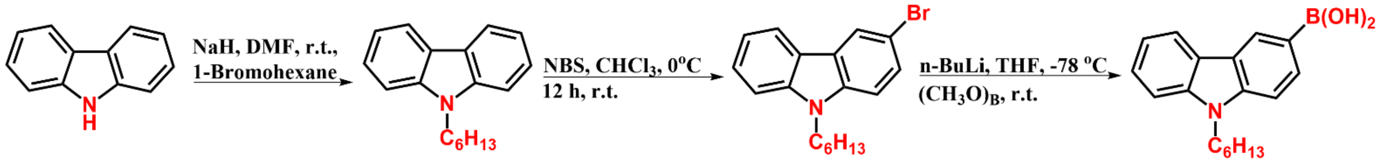

Fig. S2. Synthesis scheme of n-hexylcarbazole-3-ylboronic acid.

Synthesis of 9-hexylcarbazole (4): Carbazole (2.5 g, 15 mmol, and 1 equiv) was dissolved in 30 mL of DMF. Thereafter, NaH (1.07 g, 45 mmol, and 3 equiv) was added all at once to the reaction mixture. After the reagents were dissolved, 1-bromohexane (2.97 g, 18 mmol, and 1.2 equiv) was added dropwise. The reaction mixture was stirred overnight at room temperature. Thereafter, the reaction was quenched with water and extracted with CF three times (15 mL × 3). The organic phase was dried over anhydrous $MgSO_4$, filtered, and evaporated under reduced pressure. The product was obtained as a light yellow solid and used without further purification. (3 g; 80%) ^{1}H NMR($CDCl_3$) δ (ppm): 8.10(d, 2H), 7.49–7.40(m, 4H), 7.25(m, 2H), 4.30(m, 2H), 1.91–1.84(q, 2H), 1.41–1.30(m, 6H), 0.87(t, 3H); ^{13}C NMR: 140.48, 125.63, 122.86, 120.40, 118.75, 108.73, 43.10, 31.67, 29.00, 27.05, 22.64, and 14.12.

Synthesis of 3-Bromo-N-hexylcarbazole (5): 9-hexylcarbazole (3 g; 12 mmol) was dissolved in $CHCl_3$ (45 mL), and NBS (2.1 g; 12 mmol) was added portionwise at 0 °C. The reaction mixture was stirred at room temperature for 12 h in the dark. Afterward, water was added, and the aqueous phase was extracted with diethylether. The organic phase was dried over $MgSO_4$, filtered, and evaporated. A white solid product was obtained after column chromatography with hexane/ethylacetate (1:10). (2.5g; 75%) ^{1}H NMR($CDCl_3$) δ (ppm): 8.20(d, 1H), 8.05(d, 1H), 7.55–7.39(m, 3H), 7.29(m, 2H), 4.26(m, 3H), 1.88–1.81(q, 2H), 1.38–1.29(m, 6H), and 0.86(t, 3H); ^{13}C NMR: 140.69, 139.05, 128.21, 126.35, 124.54, 123.06, 121.80, 120.56, 119.19, 111.51, 110.14, 108.96, 43.18, 31.60, 28.91, 26.96, 22.60, and 14.09.

N-hexylcarbazole-3-ylboronic acid (6): n-BuLi (2.5 M in a hexane solution; 11.25 mmol) was added dropwise to a 3-bromo-N-hexylcarbazole solution (2.5 g; 7.5 mmol) in THF (30 mL) in a dried flask at −78 °C under a nitrogen atmosphere. After stirring for 1 h, triisopropyl borate (11.3 mmol; 2.6 mL) was added to the solution, and then the reaction mixture was warmed to room temperature and stirred overnight. The mixture was poured into a large amount of water and extracted with DCM. The organic layer was dried over $MgSO_4$ and concentrated under a vacuum. The residue was purified using silica gel column chromatography with hexane/DCM (2:1), resulting in a white solid. ^{1}H NMR ($CDCl_3$) δ (ppm): 9.09(s, 1H), 8.47(d, 1H), 8.36(d, 1H), 7.57–7.52(m, 3H), 7.47(d, 1H), 7.36(t, 1H), 4.35(t, 2H), 1.97–1.90(p, 2H), 1.38–1.31(m, 6H), and 0.90(t, 3H).

Synthesis of (4-(9H-carbazol-9-yl)phenyl)boronic acid (7):

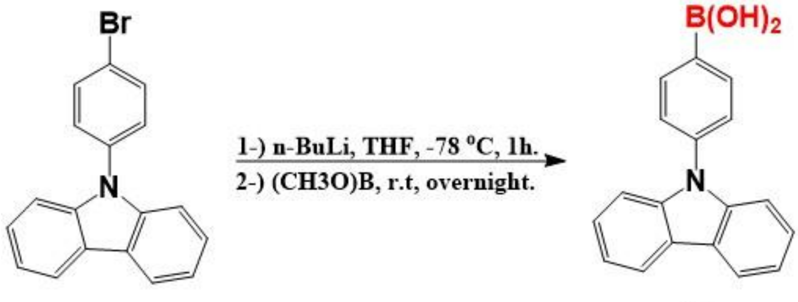

Fig. S3. One-step synthesis of 4-(9H-carbazol-9-yl)phenylboronic acid.

The same procedure was used to synthesize molecule (6).
^{1}H NMR (C_2D_6OS) δ (ppm): 8.25–8.21(m, 4H), 8.07(d, 2H), 7.57(d, 2H), 7.41(m, 4H), and 7.29–7.25(m, 2H).

Synthesis of TAT-TY1

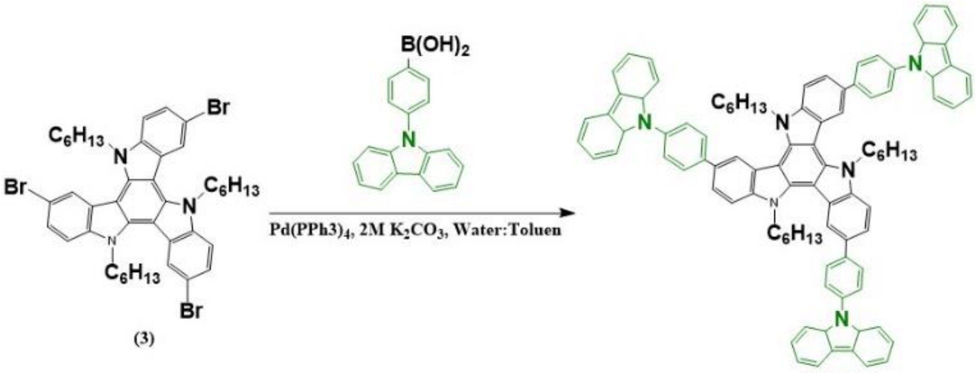

Fig. S4. Synthesis of TAT-TY1 using the triple Suzuki–Miyaura cross-coupling reaction.

To a degassed mixture of (3) (300 mg, 0.36 mmol, and 1 equiv) and 4-(9H-carbazol-9-yl)phenylboronic acid (7) (361.7 mg; 1.26 mmol) in 15 mL of toluene, 2M aqueous K_2CO_3 (2 mL) and $Pd(PPh_3)_4$ (80 mg; 18%) were added under an argon atmosphere and the reaction mixture was heated to 70 °C overnight. After being cooled to room temperature, the reaction mixture was poured into water and extracted with $CHCl_3$. The organic phase was dried over anhydrous $MgSO_4$ and concentrated under reduced

pressure. The crude product was purified using silica gel column chromatography, gradiently increasing the polarity from 4:1 v/v (hexane: CF) to 1:4 v/v (hexane:CF) in order to obtain a 198 mg mustard yellow powder (66%). ^{1}H NMR ($CDCl_3$) δ (ppm): 8.2 (d, 5H), 8.01(d, 4H), 7.95(s, 2H), 7.73(d, 6H), 7.55(t, 7H), 7.46(t, 6H), 7.32(t, 6H), 5.08(t, 6H), 2.15(m, 6H), 1.33(m, 22H), and 0.83(t, 8H); ^{13}C NMR: 141.7, 141.0, 139.6, 136.6, 135.2, 128.8, 127.5, 126.1, 123.5, 123.0, 122.0, 120.5, 120.1, 119.3, 110.0, 108.9, 103.3, 47.1, 31.6, 30.0, 26.5, 22.6, and 14.1. MS (QTOF–ESI–MS), $[M + H]^+$ calculated = 1321.6835; observed = 1321.71195 *m*/*z*.

Synthesis of TAT-TY2

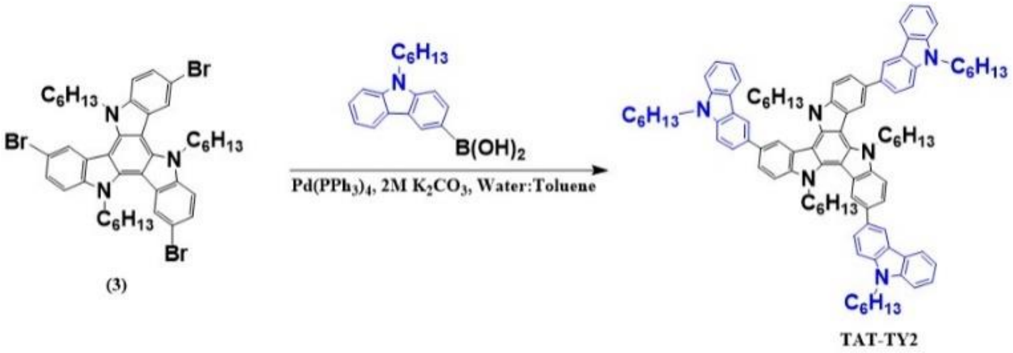

Fig. S5. Synthesis of TAT-TY2 using the triple Suzuki–Miyaura cross-coupling reaction.

The same procedure was used to synthesize TAT-TY1.

^{1}H NMR ($CDCl_3$) δ (ppm): 8.51(s, 3H), 8.45(s, 3H), 8.2(t, 5H), 7.54–7.43(m, 11H), 4.35(t, 12H), 1.92(m, 10H), 1.44–1.09(m, 27H), and 0.88(m, 40H); ^{13}C-NMR: 141.0, 140.0, 139.6, 133.4, 125.7, 125.6, 123.7, 123.4, 123.1, 120.5, 119.1, 119.0, 118.7, 109.0, 108.9, 108.8, 43.3, 32.0, 31.7, 29.8, 29.4, 29.1, 27.1, 22.8, 22.6, 14.2, and 14.1. MS (QTOF–ESI–MS) $[M + 3H]^+$ calculated = 1347.8870; observed = 1347.8635 *m*/*z*.

Perovskite precursor solution: The triple-cation perovskite precursor solutions were deposited from a precursor solution containing PbI_2 (470.23 mg), $PbBr_2$ (66.06 mg), MABr (18.81 mg), FAI (167.15 mg), and CsI (15.59 mg) in anhydrous DMF:DMSO 4:1 (v:v) into the glove box using a single-step deposition method [9].

Device fabrication: (FTO/Li-treated c-TiO_2/perovskite/HTM/MoO_3/Ag): The FTO-coated glass substrates were cleaned with deionized water, acetone, and isopropyl alcohol in an ultrasonic bath for 20 min at 50 °C in each step, respectively. The substrates were exposed to oxygen plasma for 7 min to eliminate any remaining organic impurities. A compact TiO_2 (c-TiO_2) solution was synthesized using the sol–gel method. A solution containing 5.6 mL of isopropyl alcohol, 35 μL of hydrochloric acid, and 369 μL of titanium(IV) isopropoxide was spin-coated on FTO at 2000 rpm with a ramp speed of 2000 rpm/s acc for 20 s, followed by annealing at 460 °C for 1 h on a hot plate under ambient conditions. Thereafter, a 0.1 M solution of LiTFSI in an acetonitrile layer was deposited on c-TiO_2 by spin-coating at 3000 rpm with a ramp speed of 2000 rpm/s acc for 30 s [10]. All the electrodes were calcined using a second calcination step at 460

°C for 30 min on a hot plate under ambient conditions. All the substrates were cooled to 120 °C and then transferred to a nitrogen-filled glove box. The perovskite solution was spin-coated at 2500 rpm for 10 s with a ramp speed of 500 rpm/s acc and then at 5000 rpm for 20 s with the same acc in the glove box. At the second step, anhydrous 100 μL of CB was dropped on the center of the substrates for 10 s. The spin-coated perovskite films were then annealed at 100 °C for 1 h on a hot plate in the glove box. For the control cell, an HTM solution containing 73 mg of spiro-OMeTAD in 1 mL of anhydrous CB was used with 16 μL of a LiTFSI/anhydrous acetonitrile solution (520 mg/mL) and 30 μL of tBP for doping. The HTM was spin-coated on the perovskite at 4000 rpm with 500 rpm/s acc for 20 s[9]. An HTM solutions containing 60 mg of TAT-H, TAT-HBr, TAT-TY1, and TAT-TY2 molecules; 16 μL of LiTFSI; and 30 μL of tBP in CB was spin-coated on the top of the perovskite layer at a spin speed of 4000 rpm at 500 rpm/s acc for 20 s. Thereafter, 8 nm MoO_3 and 80 nm silver top electrodes were deposited by high-vacuum thermal evaporation at 2×10^{-7} Torr through a shadow mask with a device area of 0.016 cm^2. The device areas were measured with and aperture of 0.095 cm^2. The dark and light current density-voltage characteristics of the cells (J-V) were obtained using a Keithley 2400 source meter. The voltage scan rate used was typically 10 mV/s-1. An AM1.5G filter was used as the light source, and a light power of 100 mW/cm2 was employed for all solar J-V measurements. The light intensity was calibrated using a silicon solar cell with an area of 4 cm2. The incident photon-to-current efficiency (IPCE) measurements were conducted using the QE-R Enlitech device.

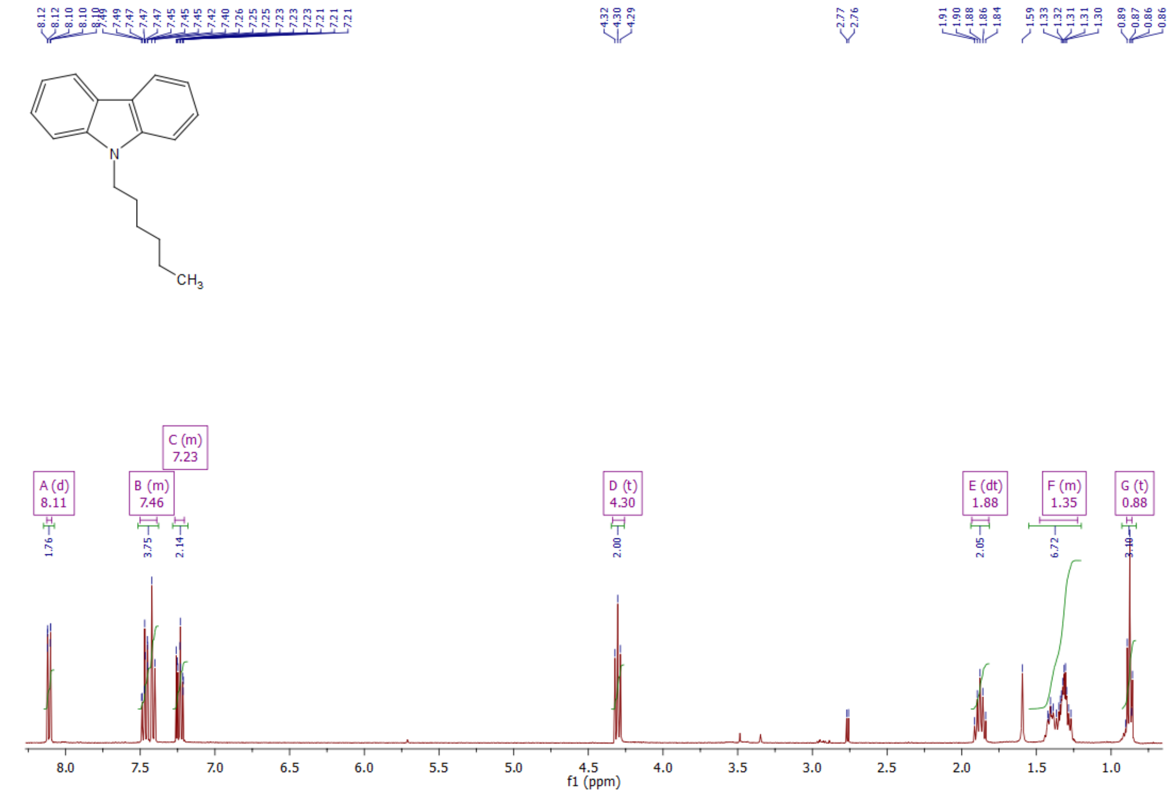

Figure S6: ^{1}H-NMR spectra of 9-hexylcarbazole in chloroform-d.

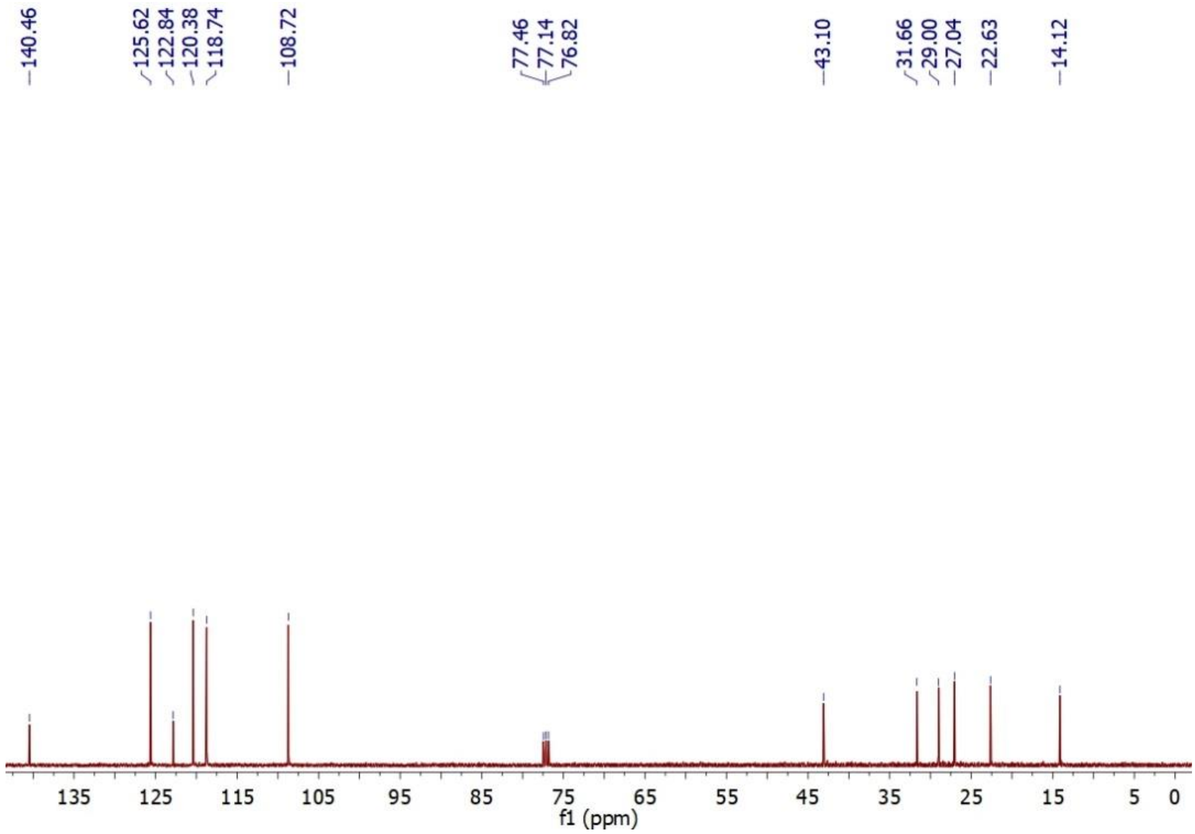

Figure S7: ^{13}C-NMR spectra of 9-hexylcarbazole in chloroform-d.

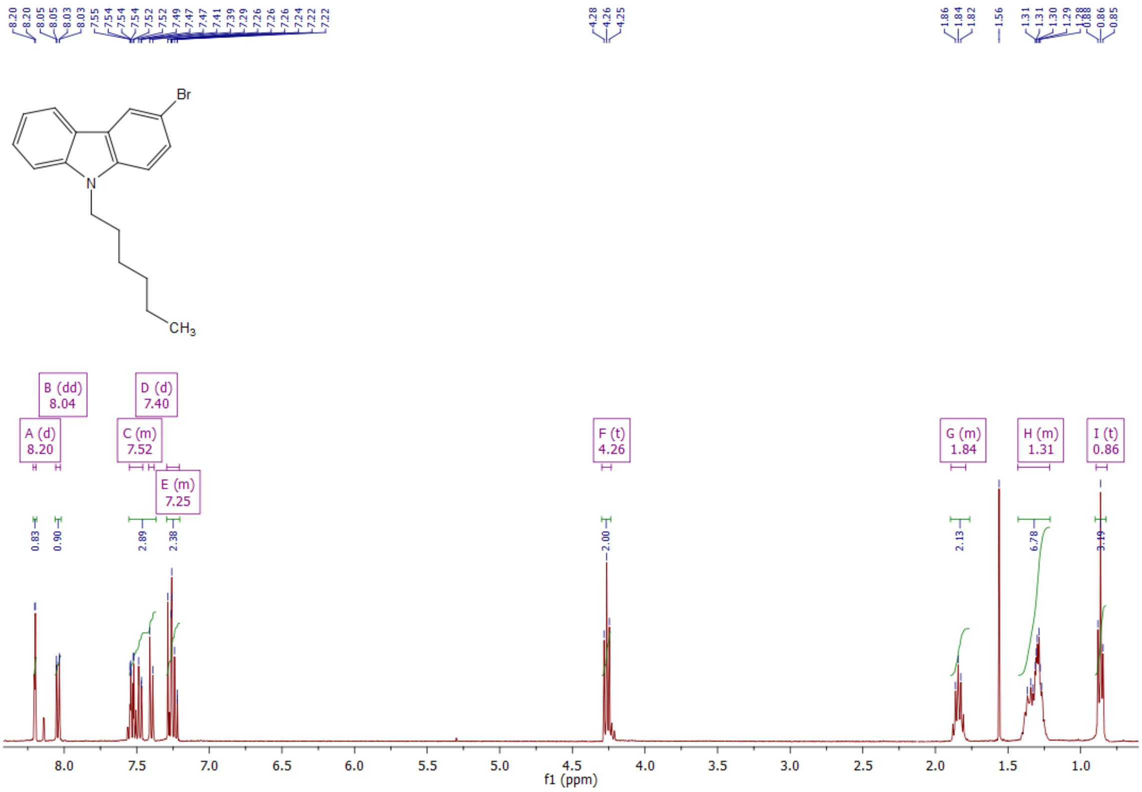

Figure S8: ^{1}H-NMR spectra of 3-bromo-N-hexylcarbazole in chloroform-d.

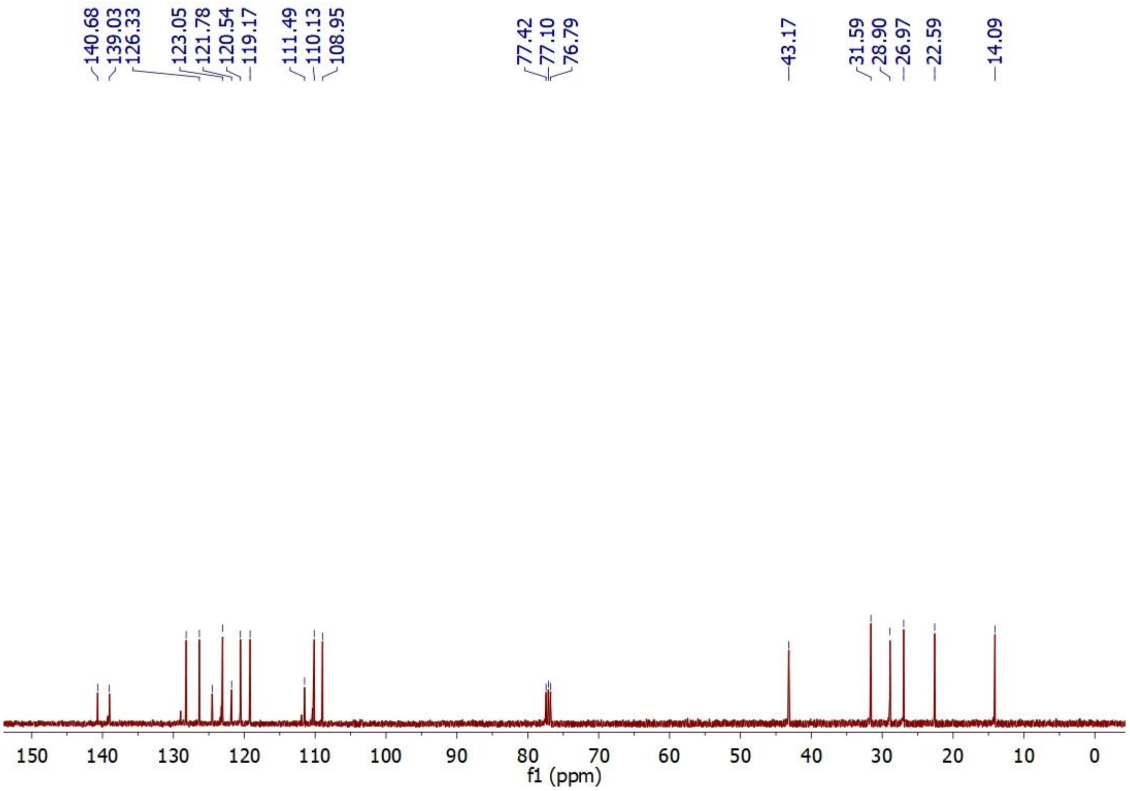

Figure S9: ^{13}C-NMR spectra of 3-bromo-N-hexylcarbazole in chloroform-d.

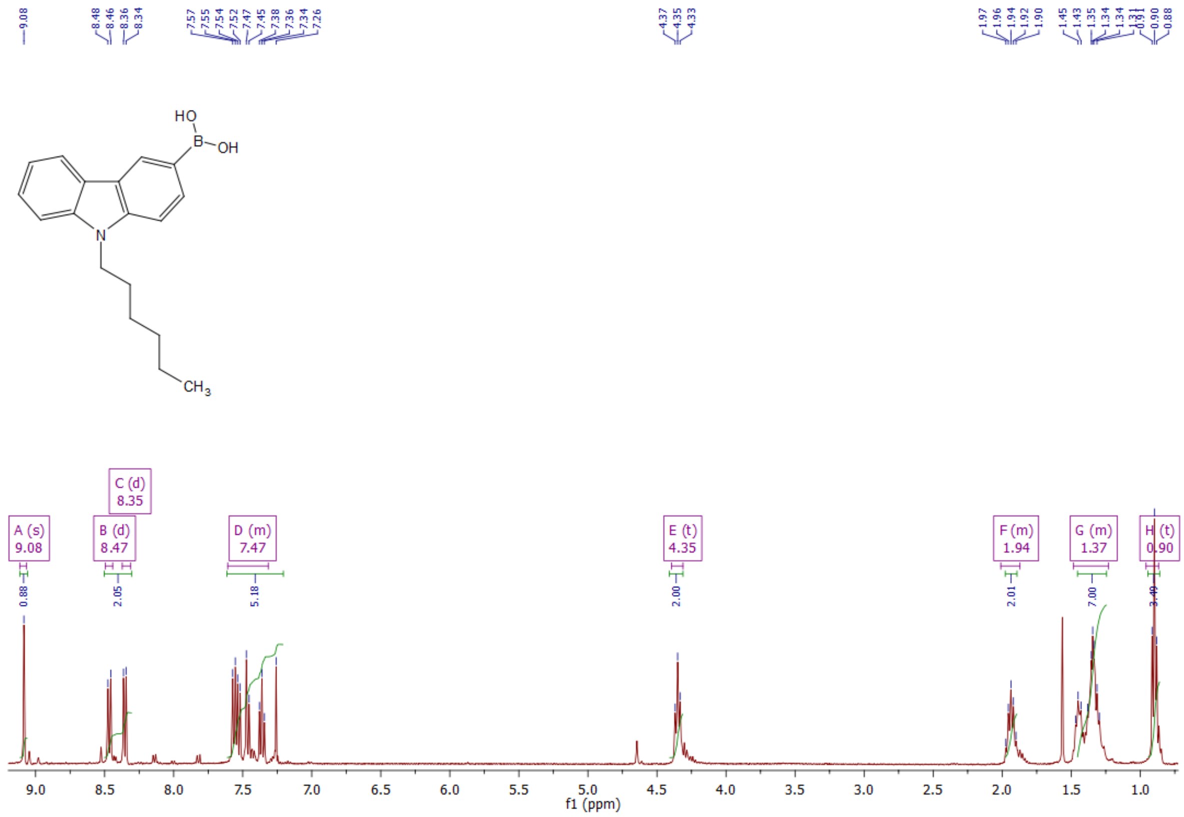

Figure S10: ^{1}H-NMR spectra of N-hexylcarbazole-3-ylboronic acid in chloroform-d

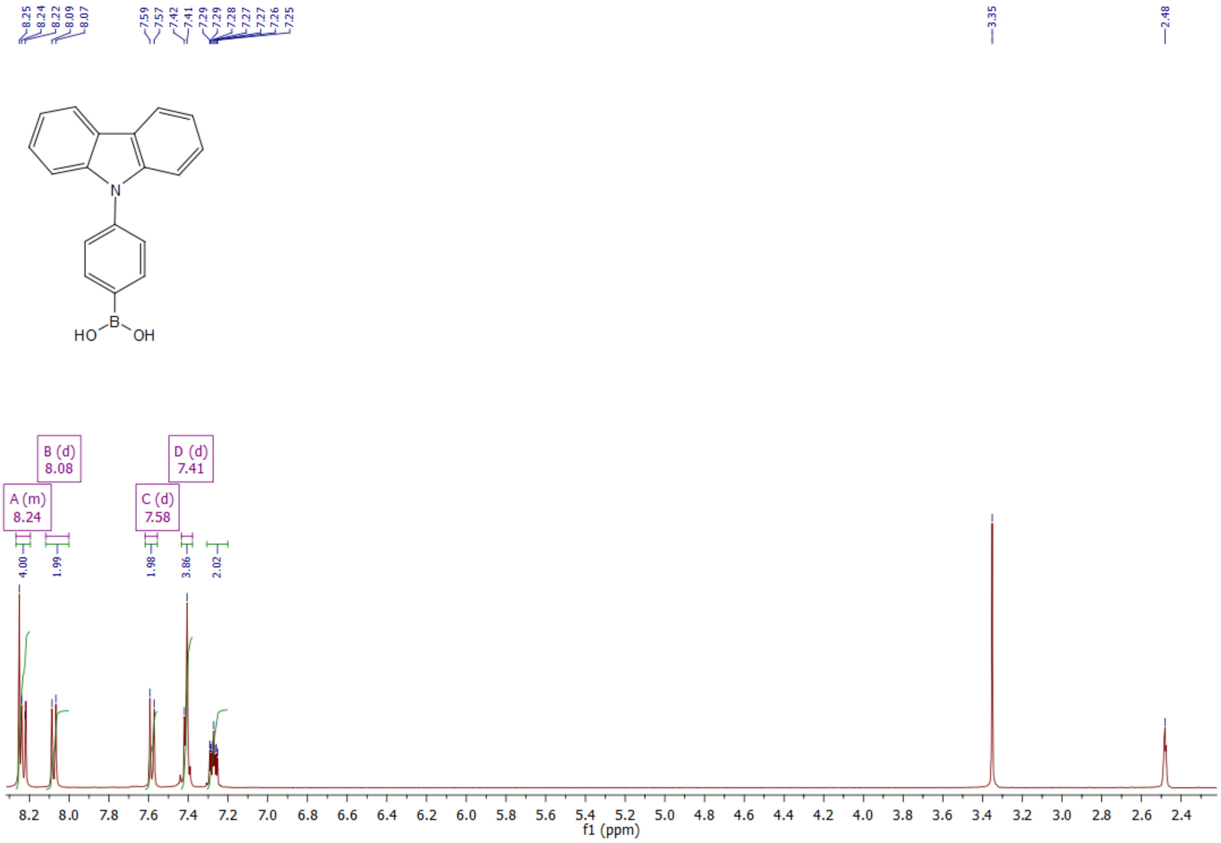

Figure S11: 1-HNMR spectra of 4-(9H-9-carbazole)phenylboronic acid in DMSO-d

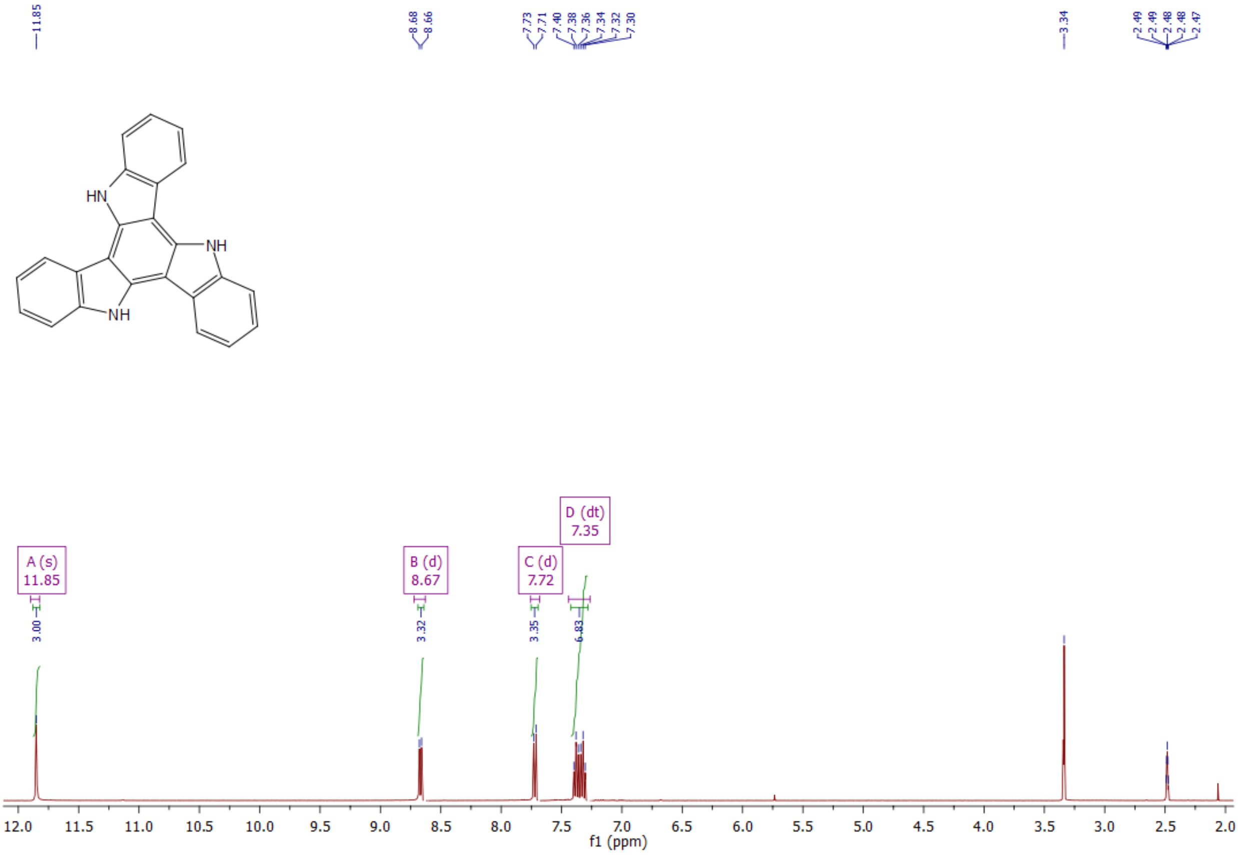

Figure S12: ^{1}H-NMR spectra of triazatruxene in DMSO-d.

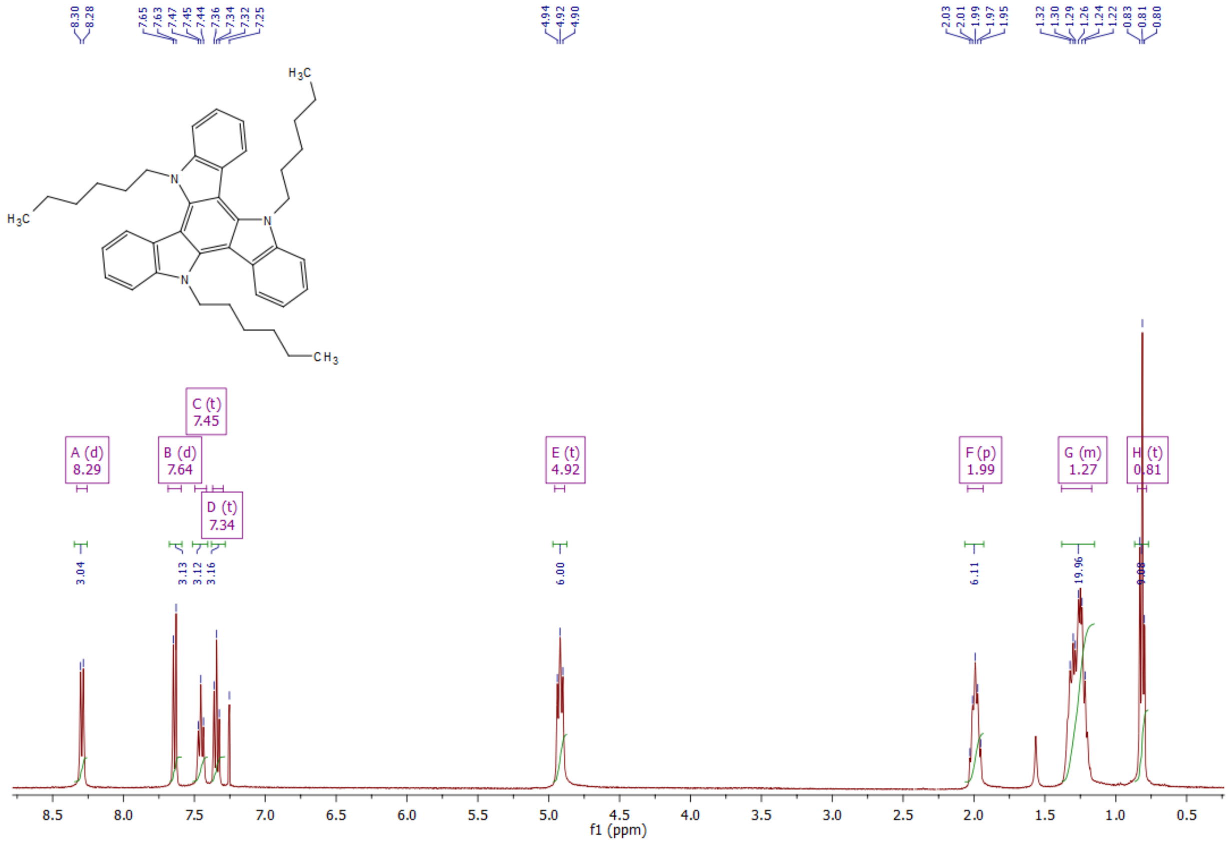

Figure S13: ^{1}H-NMR spectra of 5,10,15-trihexyl-10,15-dihydro-5H-diindolo[3,2-a:3',2'-c]carbazole in chloroform-d.

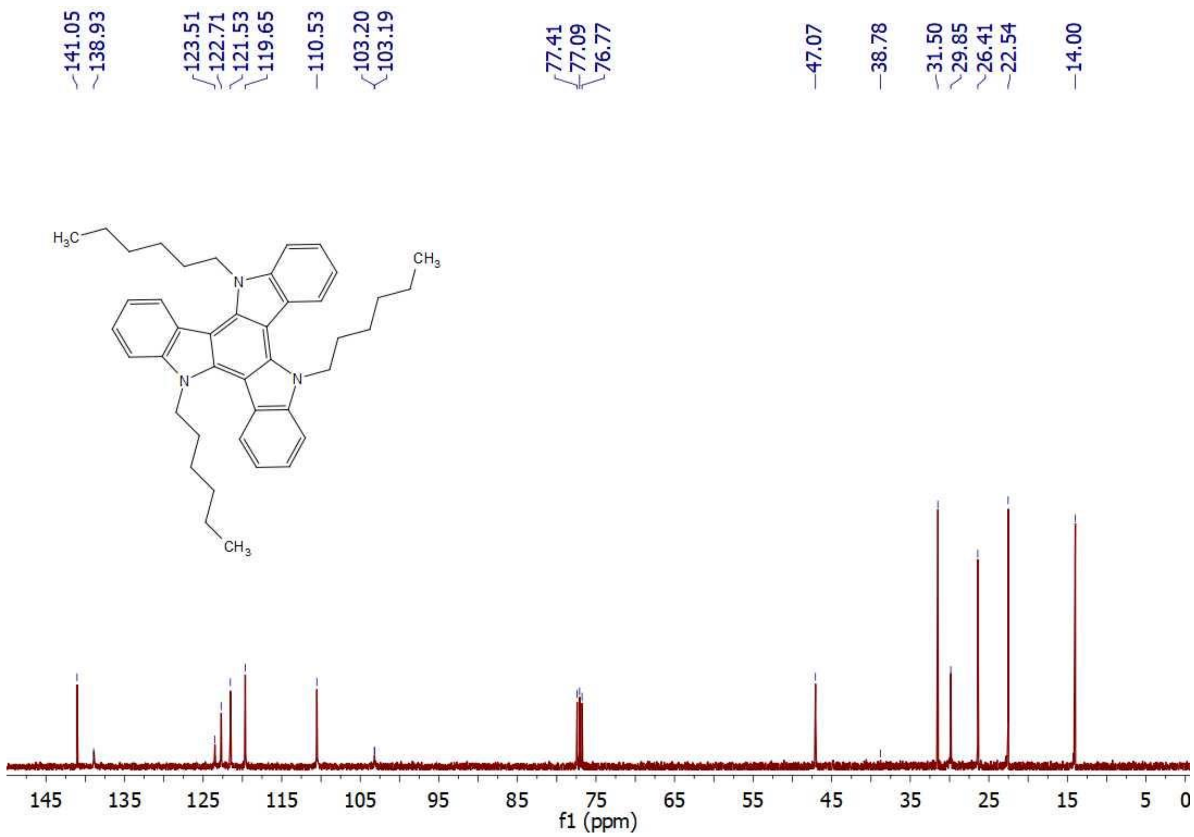

Figure S14: ^{13}C-NMR spectra of 5,10,15-trihexyl-10,15-dihydro-5H-diindolo[3,2-a:3',2'-c]carbazole in chloroform-d.

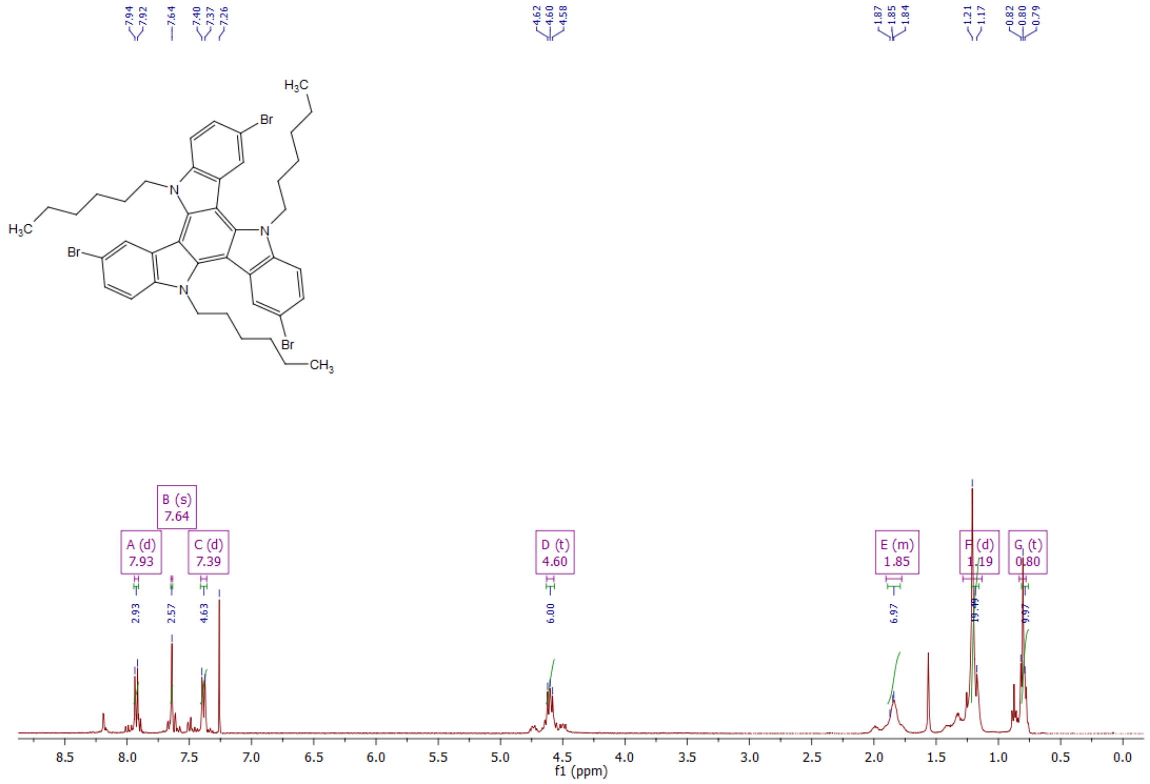

Figure S15: ^{1}H-NMR spectra of 3,8,13-tribromo-5,10,15-trihexyl-10,15-dihydro-5H-diindolo[3,2-a:3',2'-c]carbazole in chloroform-d

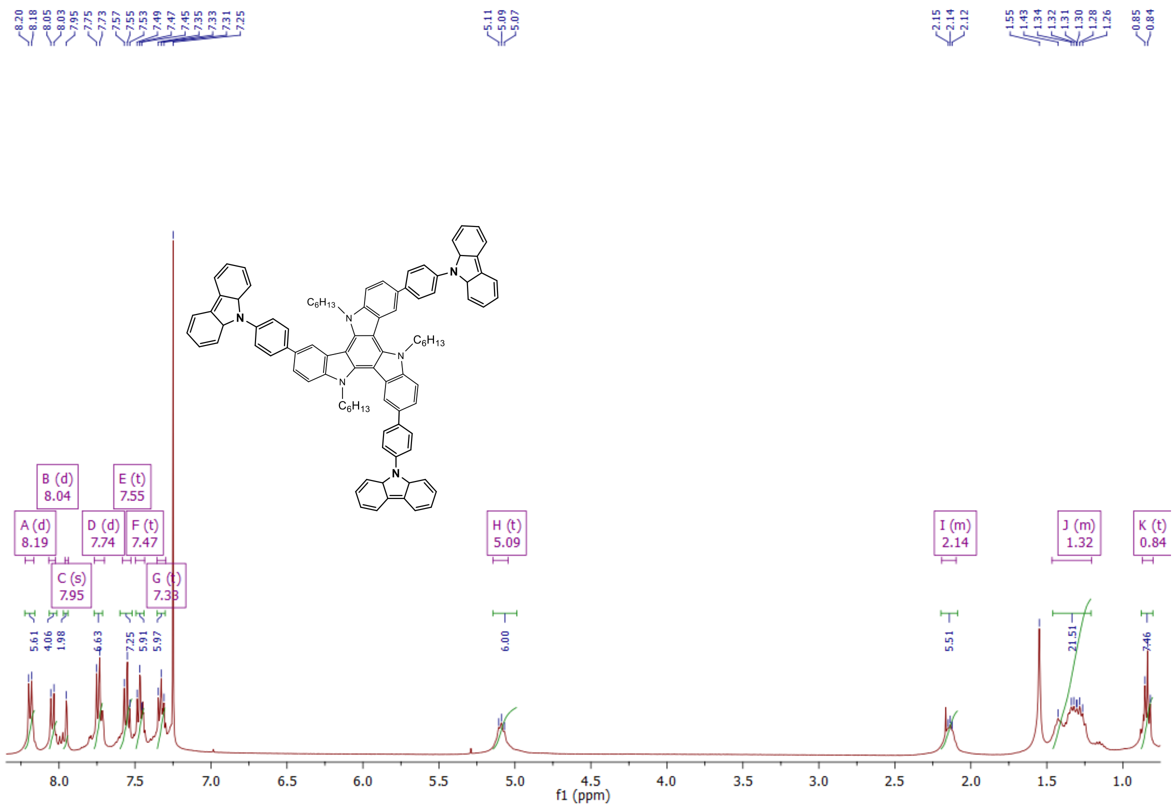

Figure S16: ^{1}H-NMR spectra of 3,8,13-tris(4-(8a,9a-dihydro-9H-carbazol-9-yl)phenyl)-5,10,15-trihexyl-10,15-dihydro-5H-diindolo[3,2-a:3’,2’-c]carbazole (TAT-TY1) in chloroform-d.

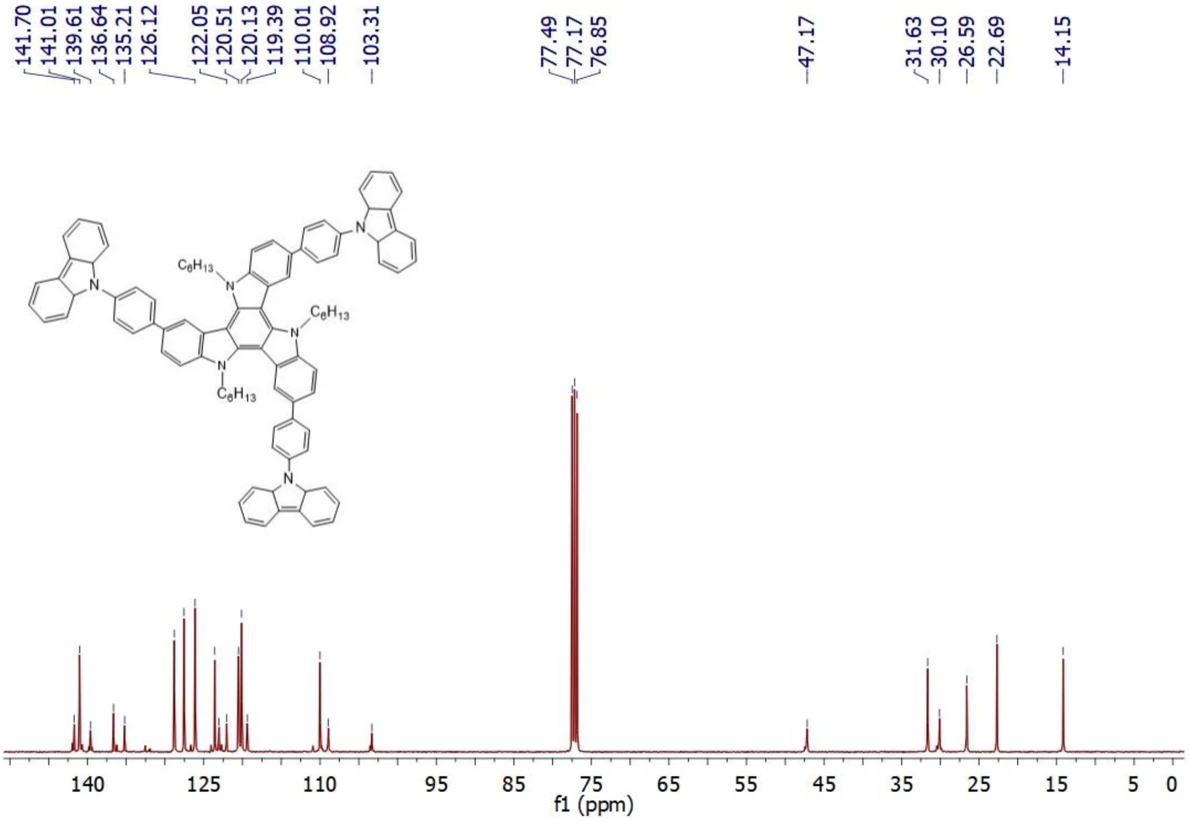

Figure S17: ^{13}C-NMR spectra of 3,8,13-tris(4-(8a,9a-dihydro-9H-carbazol-9-yl)phenyl)-5,10,15-trihexyl-10,15-dihydro-5H-diindolo[3,2-a:3’,2’-c]carbazole (TAT-TY1) *in chloroform-d*

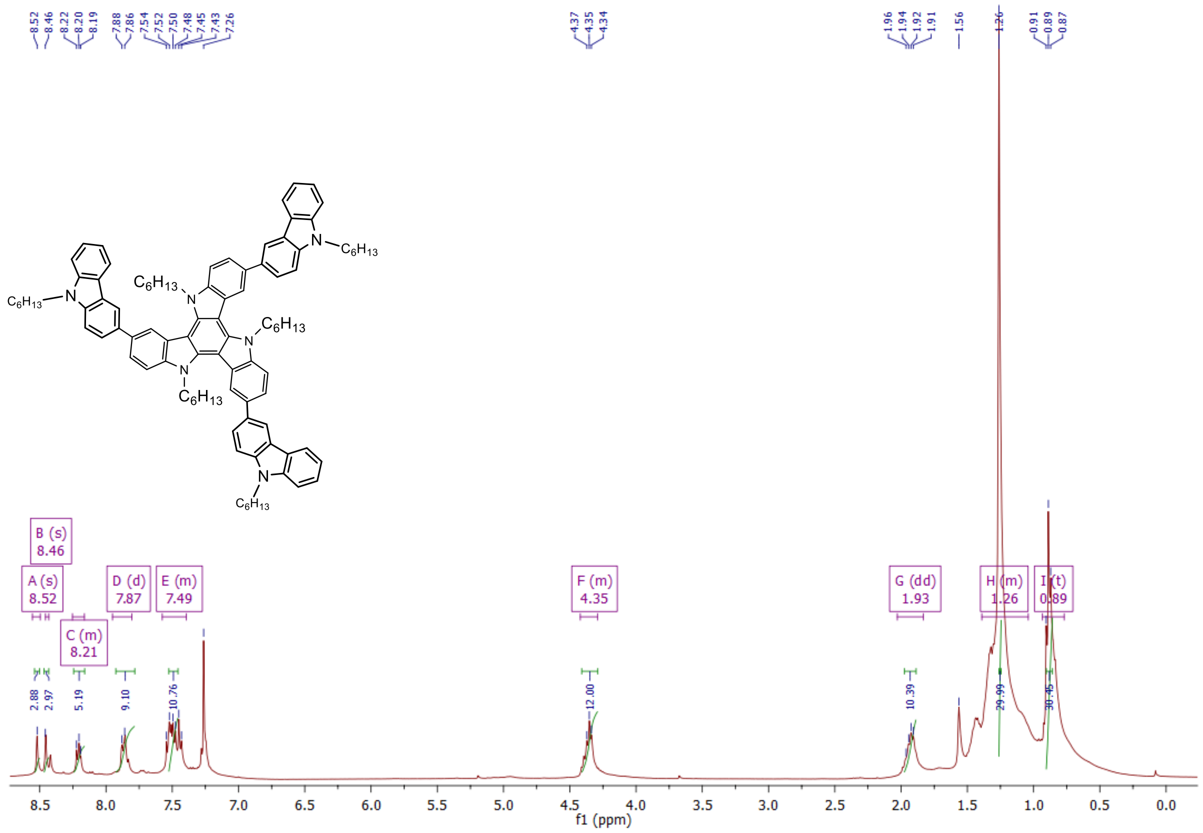

Figure S18: ^{1}H-NMR spectra of 5,10,15-trihexyl-3,8,13-tris(9-hexyl-9H-carbazol-3-yl)-10,15-dihydro-5H-diindolo[3,2-a:3',2'-c]carbazole (TAT-TY2) in chloroform-d.

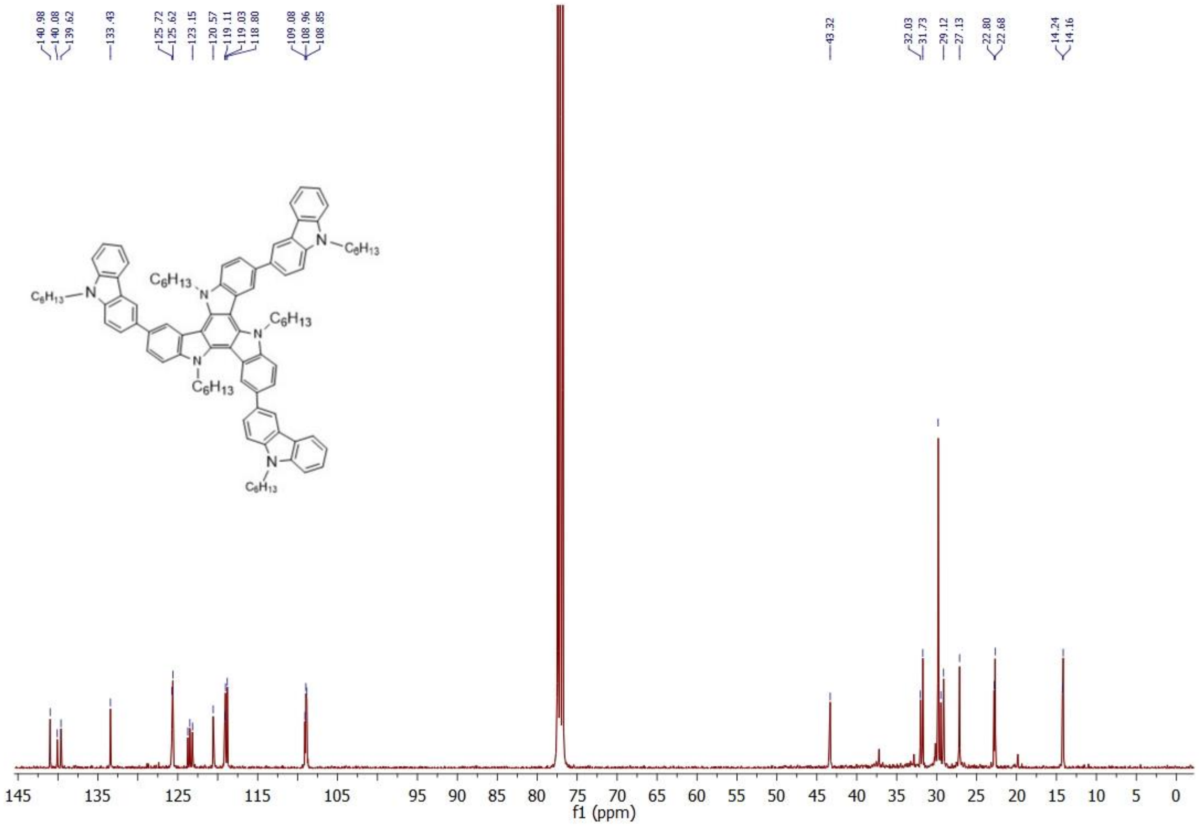

Figure S19: ^{13}C-NMR spectra of 5,10,15-trihexyl-3,8,13-tris(9-hexyl-9H-carbazol-3-yl)-10,15-dihydro-5H-diindolo[3,2-a:3',2'-c]carbazole (TAT-TY2) in chloroform-d

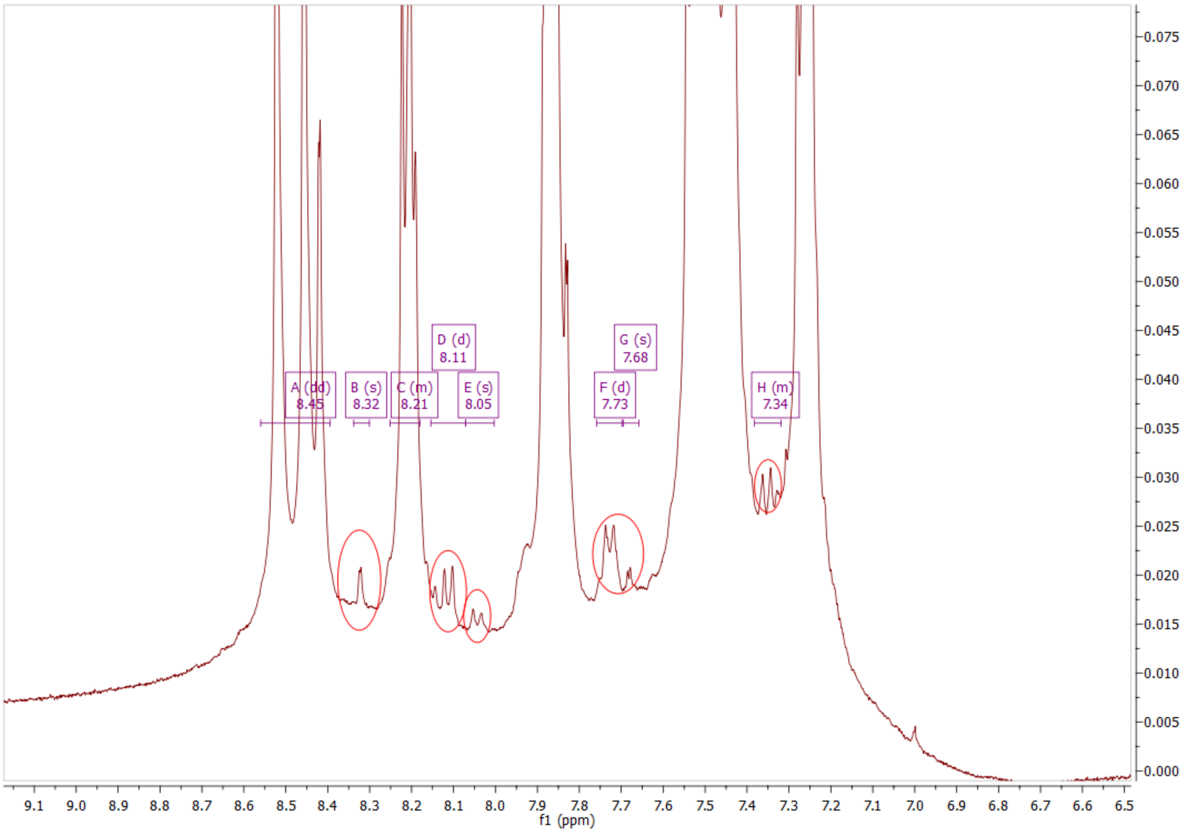

Figure S20*:* Expansion of aromatic resonance region of ^{1}H-NMR spectra corresponding to TAT-TY2 molecule and multiplet analysis. Red circles are corresponding to trace amount of impurities. Sample purity calculated by using qNMR as 98,2%.

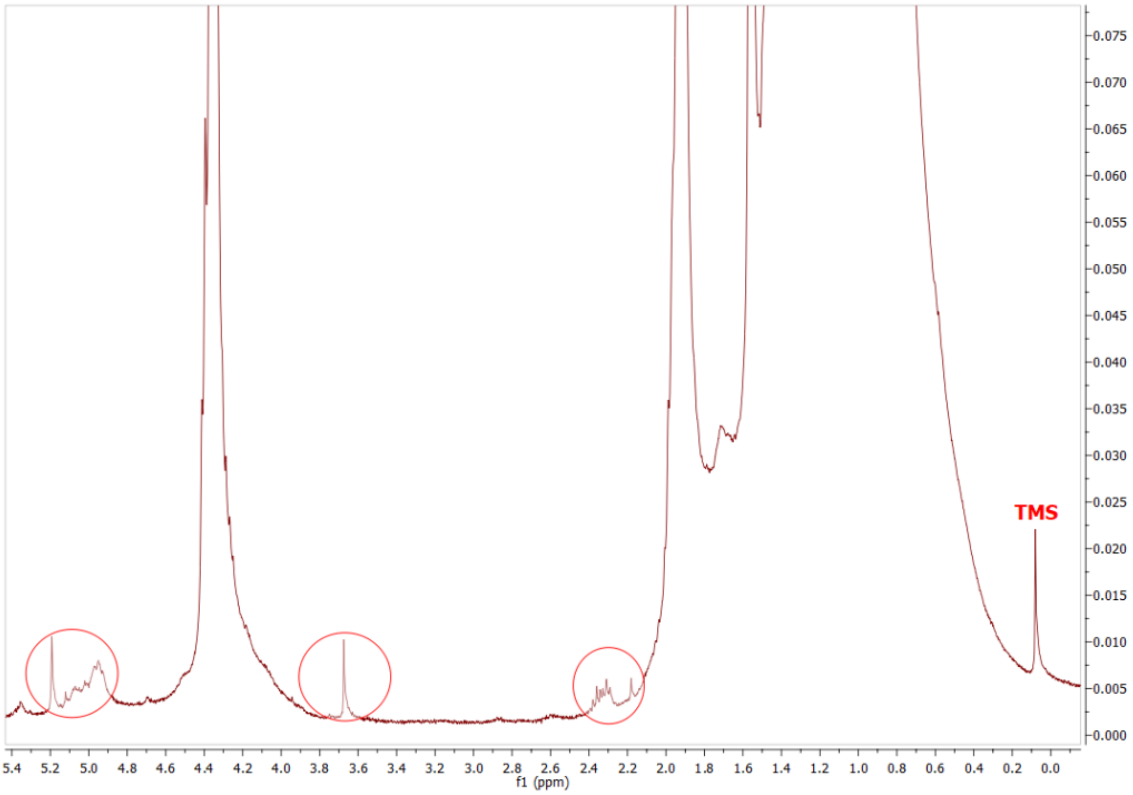

.

Figure S21: Expansion of aliphatic resonance region of ^{1}H-NMR spectra corresponding to TAT-TY2 molecule and multiplet analysis. Red circles are corresponding to trace amount of impurities. Sample purity calculated by using qNMR as 98,2%.

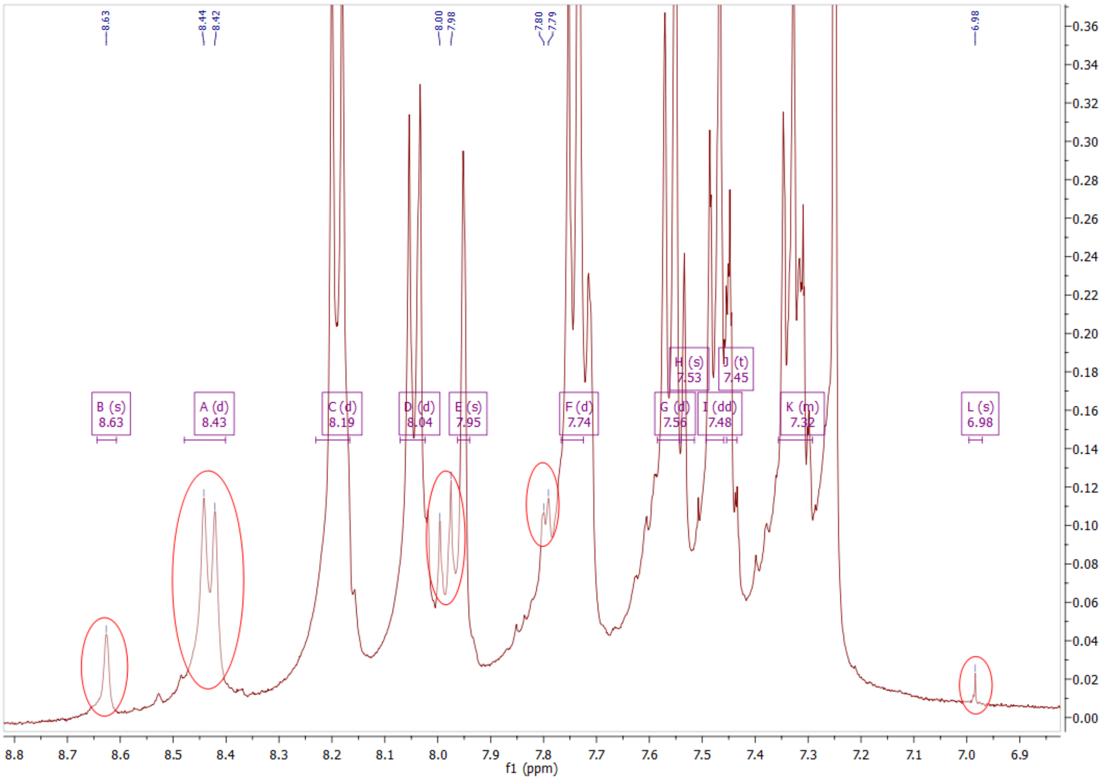

Figure S22: Expansion of aromatic resonance region of ^{1}H-NMR spectra corresponding to TAT-TY1 molecule and multiplet analysis. Red circles are corresponding to trace amount of impurities. Sample purity calculated by qNMR as 96,5%.

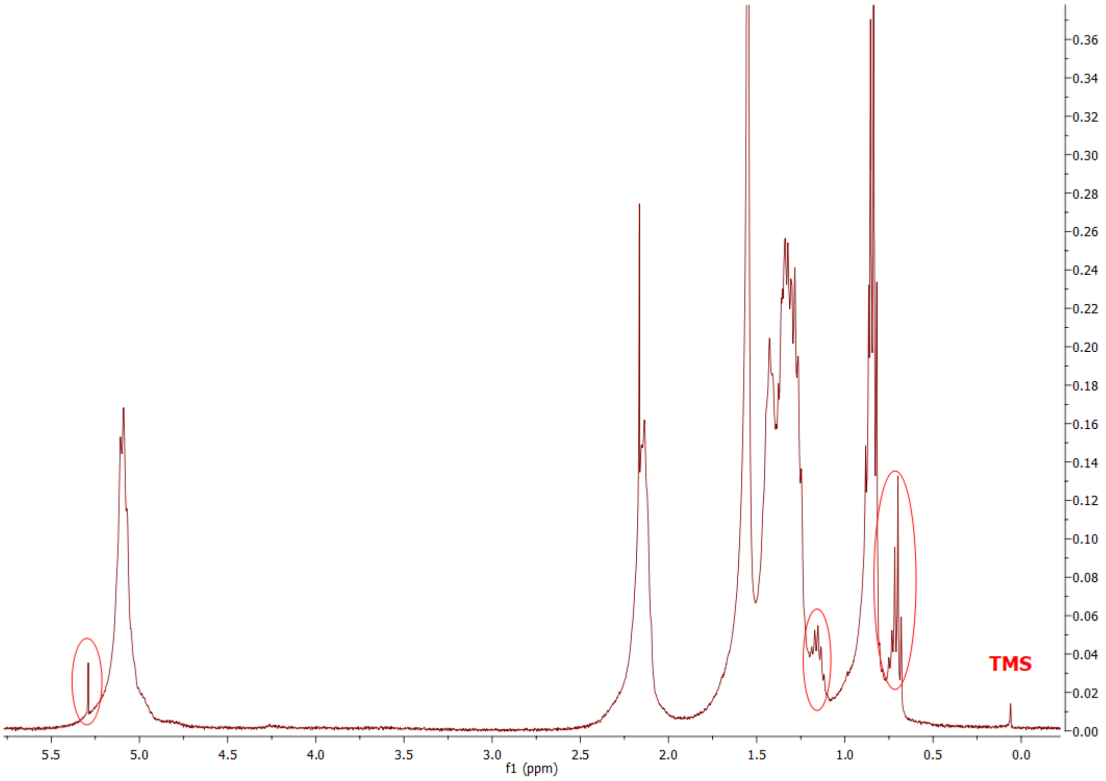

Figure S23: Expansion of aliphatic resonance region of ^{1}H-NMR spectra corresponding to TAT-TY1 molecule and multiplet analysis. Red circles are corresponding to trace amount of impurities. Sample purity calculated by qNMR as 96,5%.

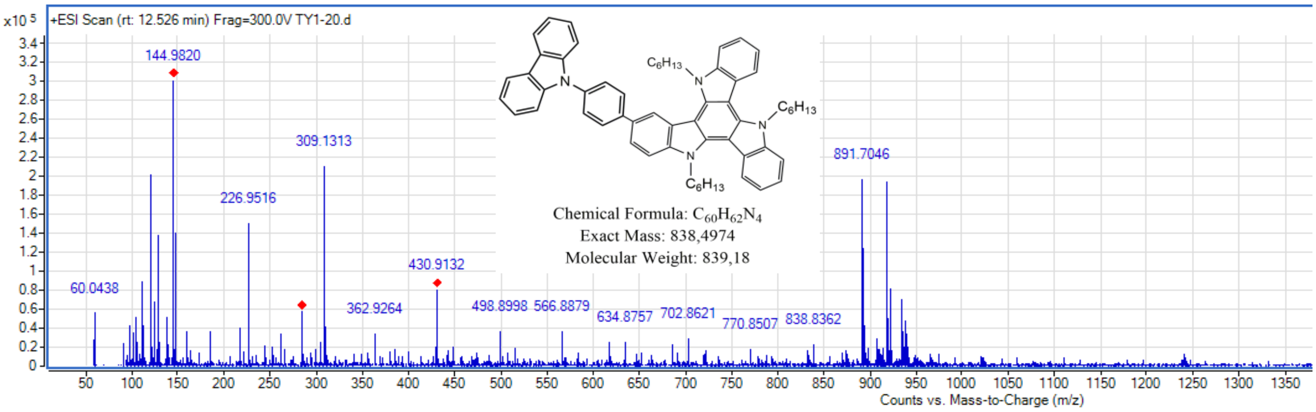

Figure S24: HRMS spectra of fragmentated TAT-TY1 molecule.

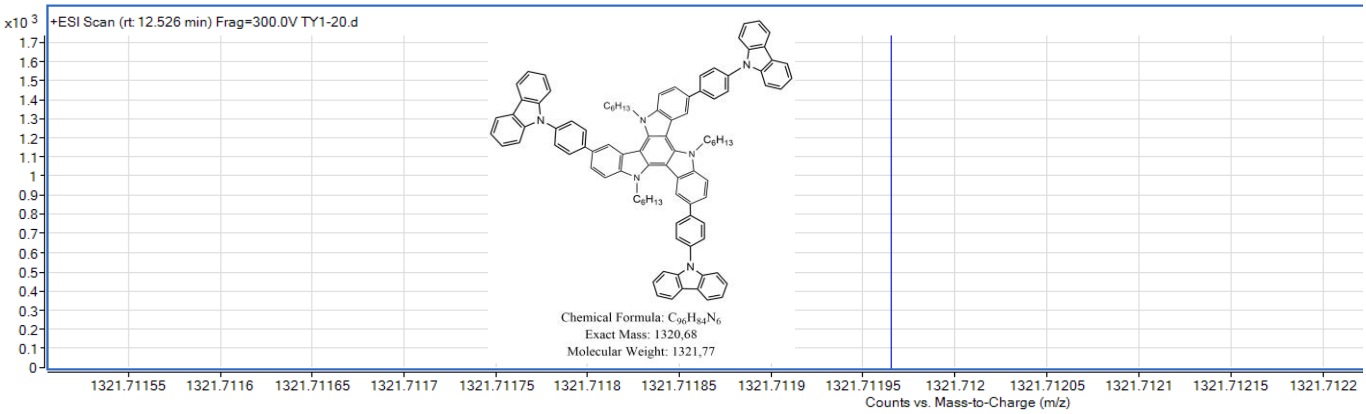

Figure S25: HRMS spectra of protonated molecular ion of TAT-TY1 $[M+1H]^{+}$.

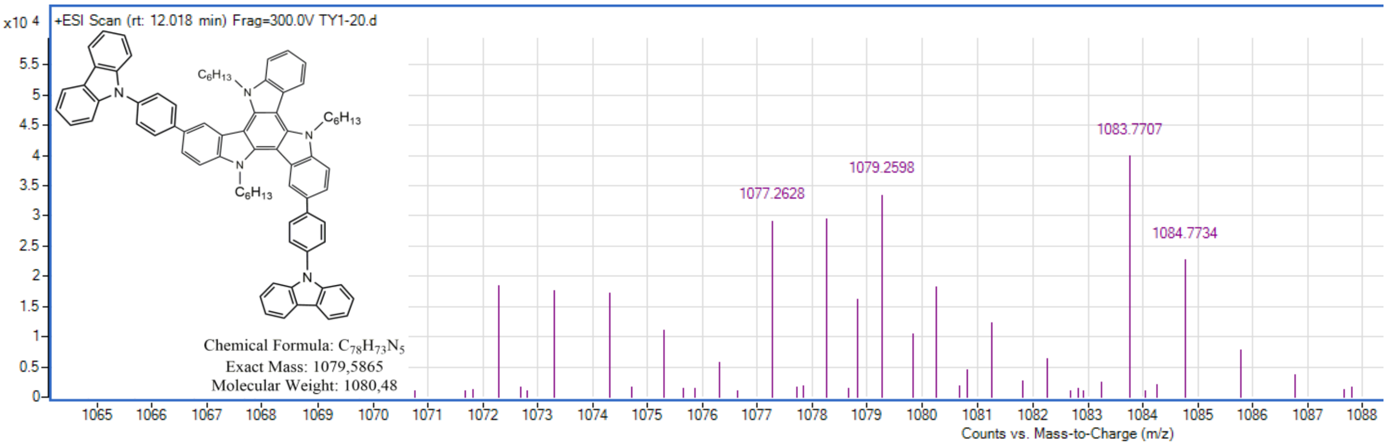

Figure S26: HRMS spectra of fragmentated TAT-TY1 molecular ion.

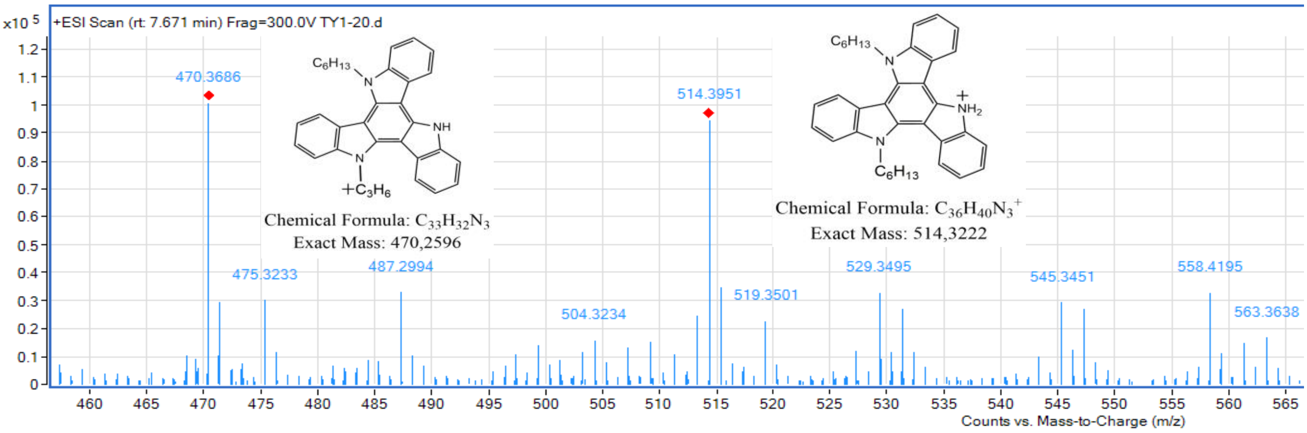

Figure S27: HRMS spectra of ionized main core (TAT-H) obtained from analysis of TAT-TY1

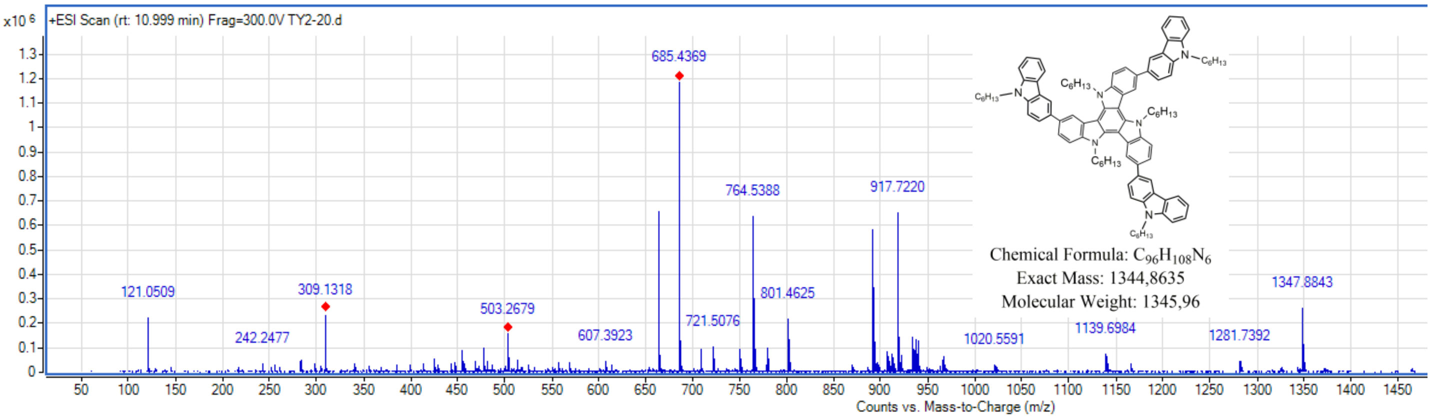

Figure S28: HRMS spectra of protonated molecular ion of TAT-TY2 $[M+3H]^+$.

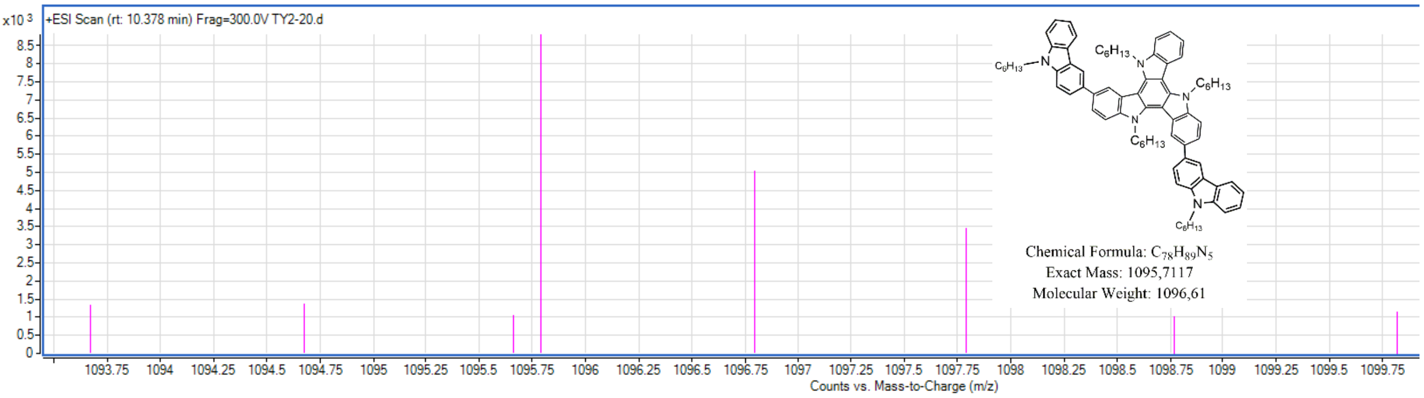

Figure S29: HRMS spectra of fragmentated TAT-TY2 molecular ion.

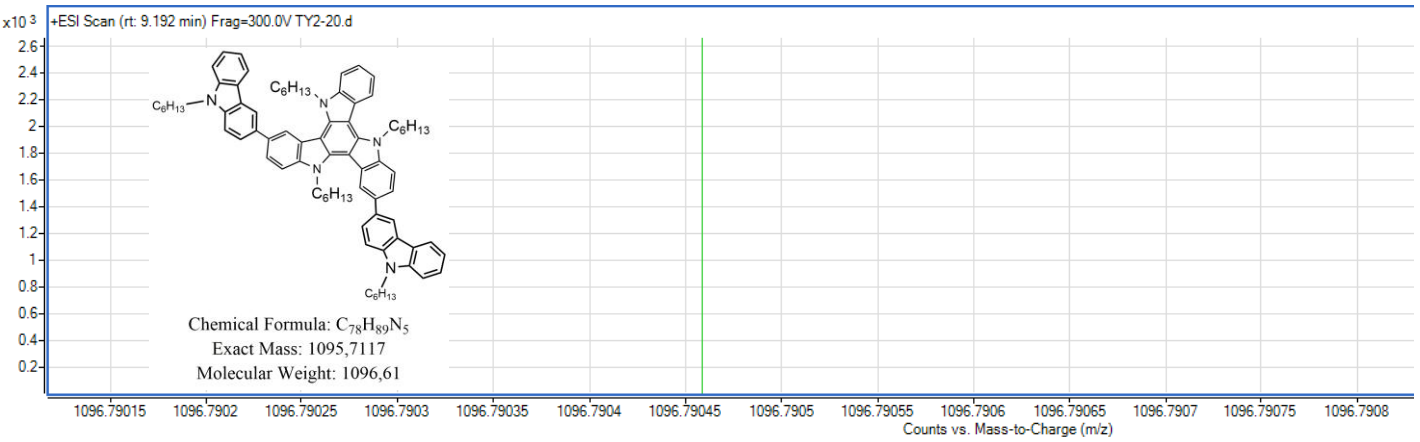

Figure S30: HRMS spectra of fragmentated TAT-TY2 molecular ion.

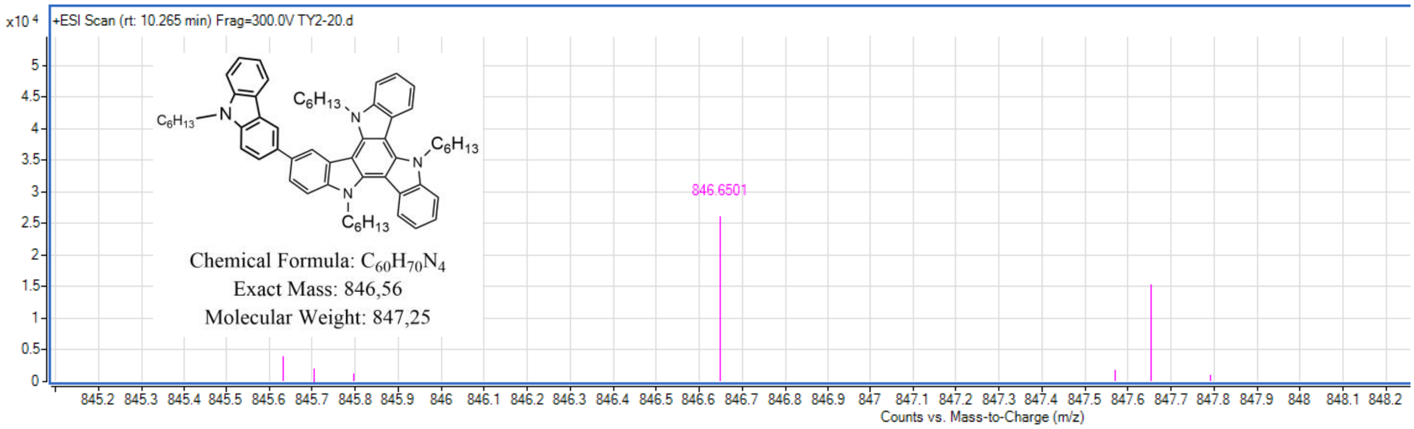

Figure S31: HRMS spectra of fragmentated TAT-TY2 molecular ion.

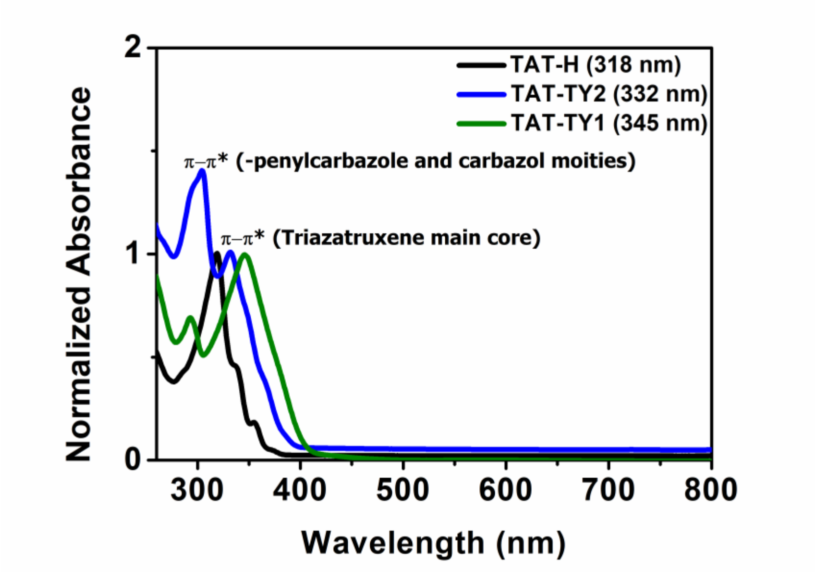

Figure S32: Full absorption spectrum of TAT-H, TAT-TY2 and TAT-TY1 including π-π* energy transitions of n-hexyl carbazol and n-phenyl carbazole moities.

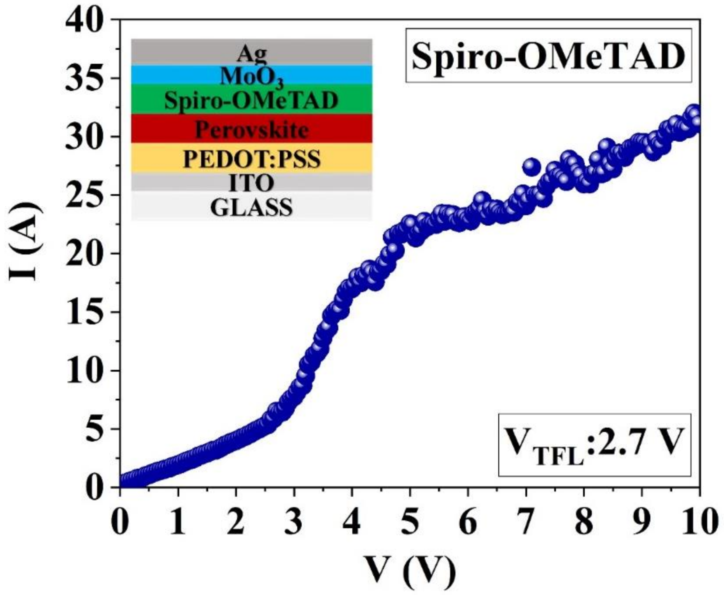

Figure S33. Space charge-limited current (SCLC) measurements of the hole-only devices corresponding to Spiro-OMeTAD (the inset showing the device structures).

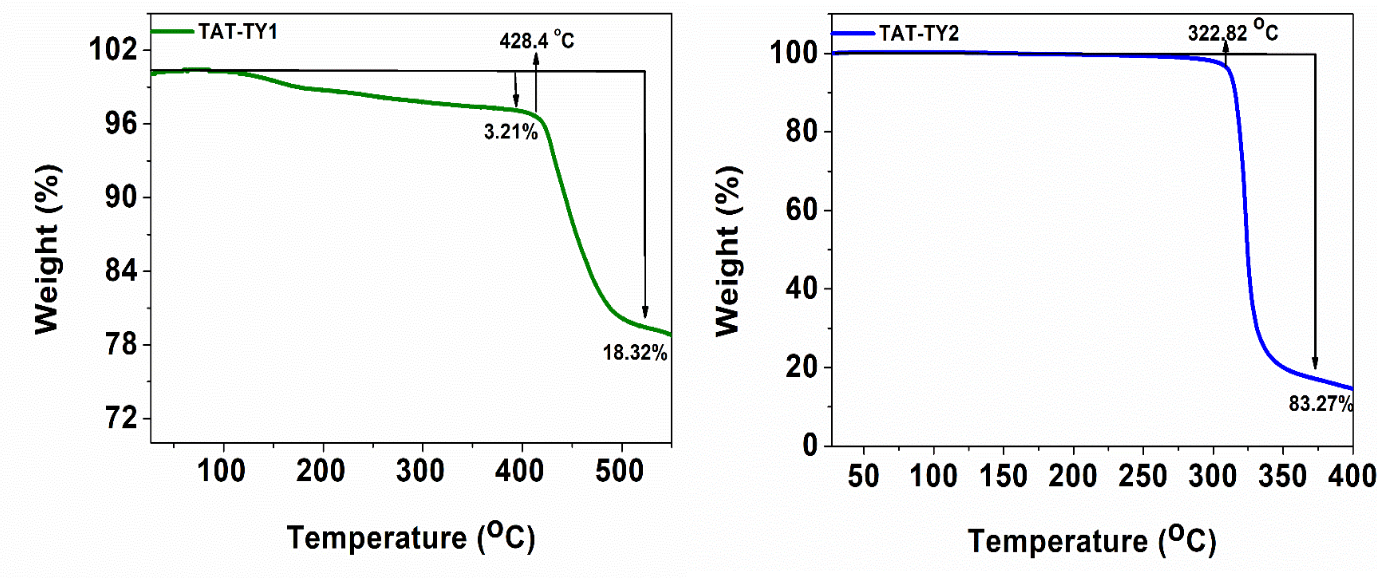

Figure S34: Termograms corresponding to TGA analysis of TAT-TY1 and TAT-TY2.

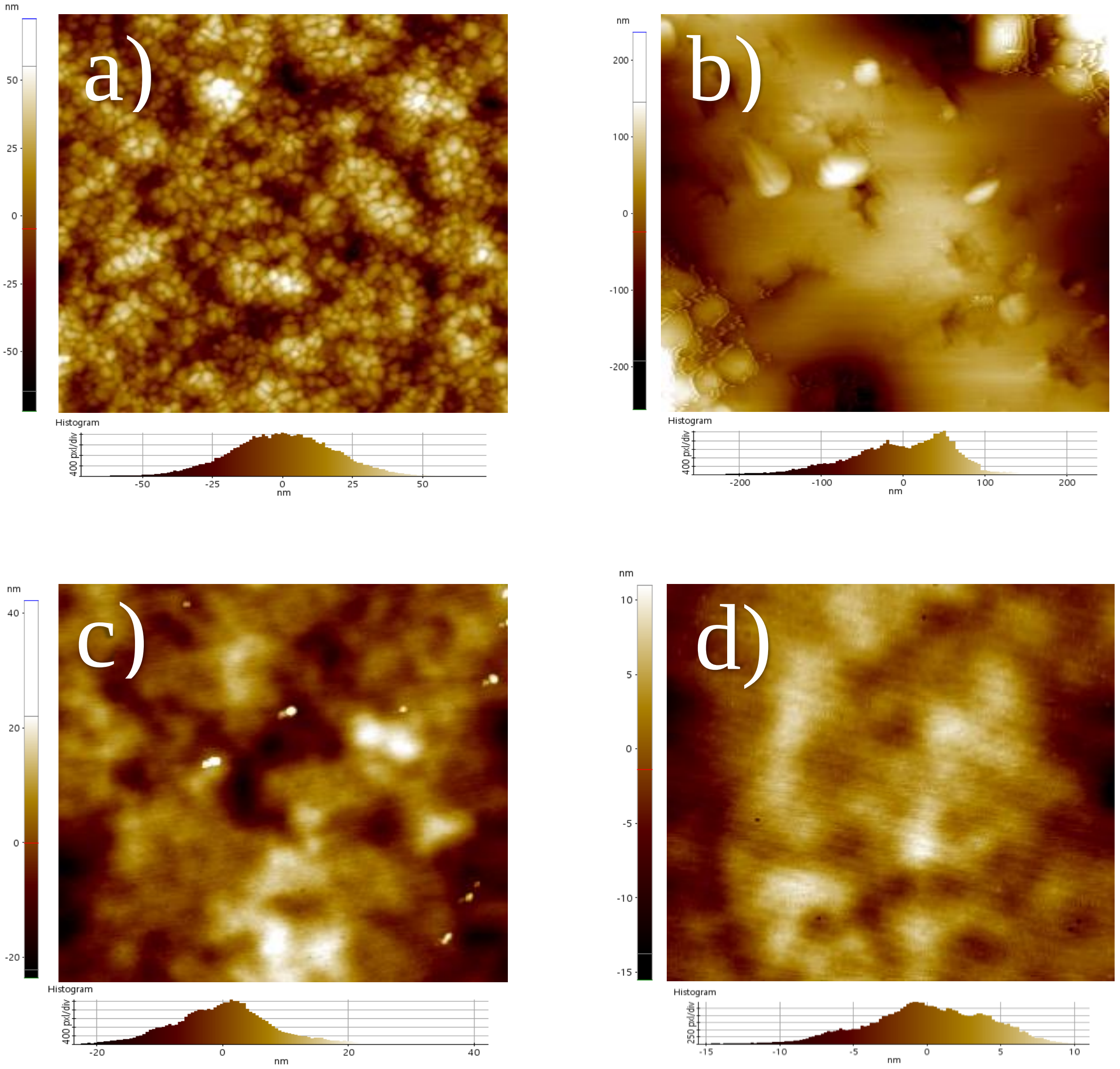

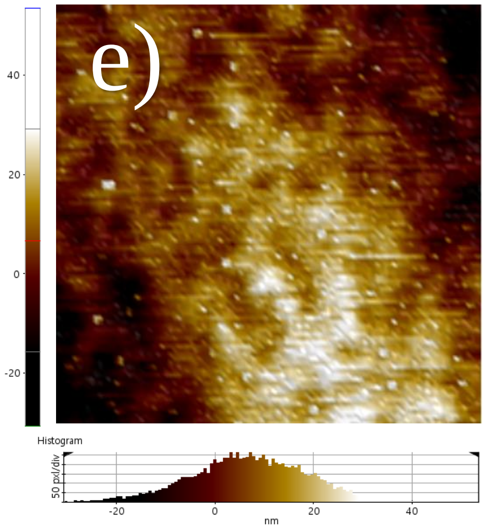

Figure S35: AFM images of bare perovskite (a, RMS: 16.7 nm), perovskite/TAT-H (b, RMS: 58.8 nm), perovskite/TAT-TY2 (c, RMS: 2.9 nm), perovskite/TAT-TY1 (d, RMS: 6.2 nm) and (e) perovskite/Spiro-OMeTAD (RMS: 14.1 nm) on glass surface.

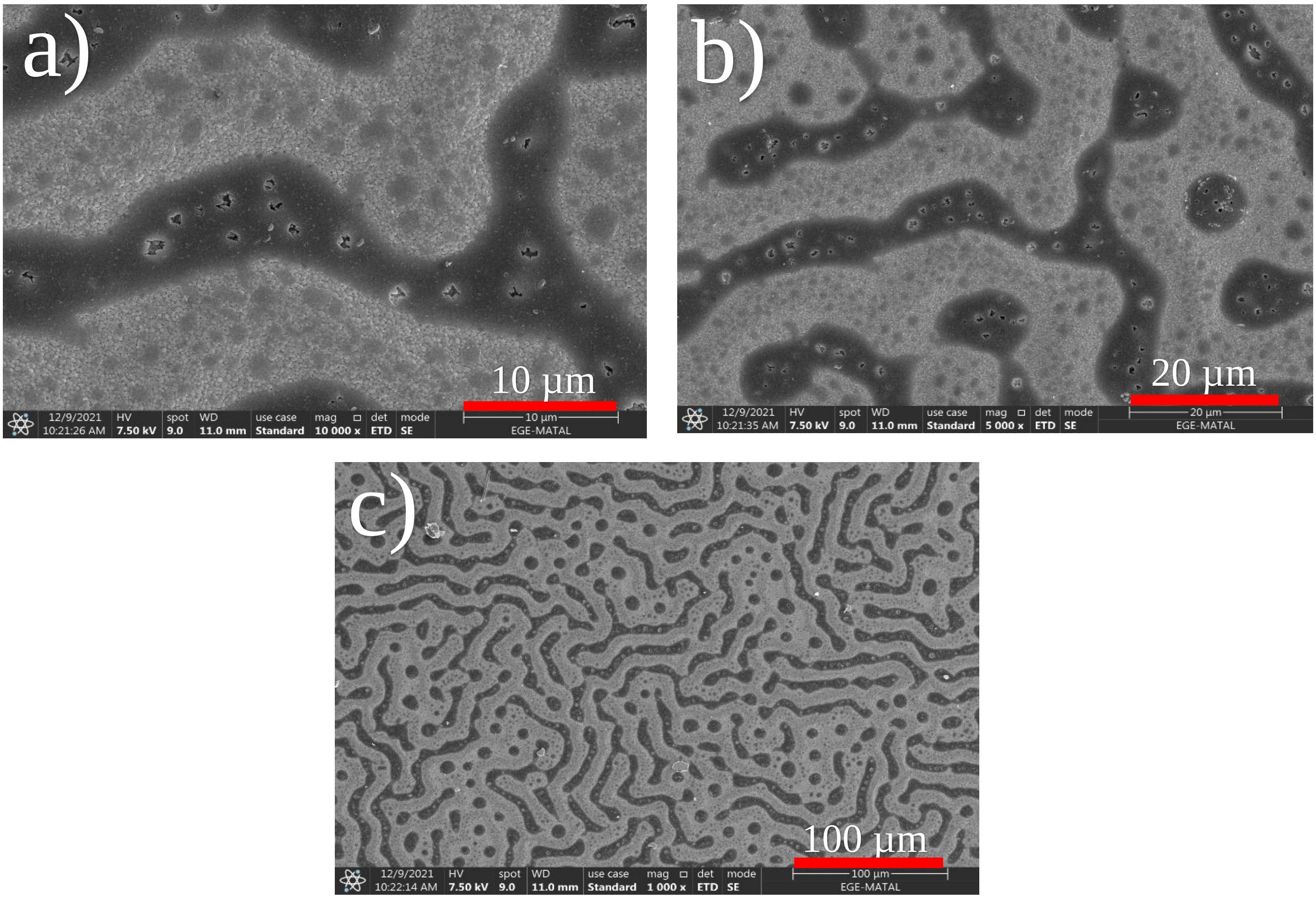

Figure S36: SEM images of TAT-H molecule on top of the perovskite layer ranging 10µm (a), 20 µm (b) and 100 µm (c). Light gray areas represents the perovskite grains

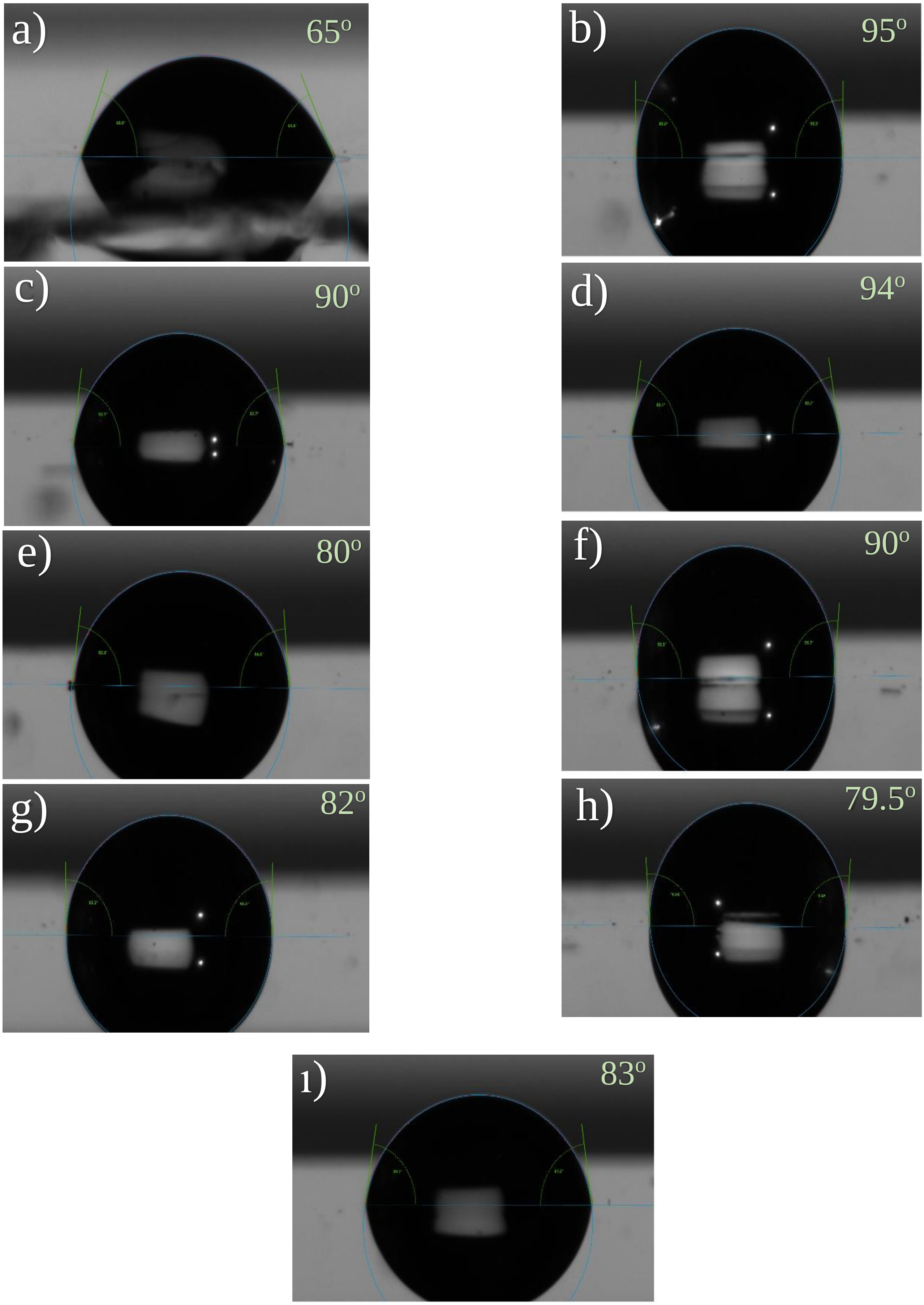

Figure S37: Contact angles of Perovskite (a) doped TAT-H (b), Spiro-OmeTAD (c), TAT-TY1 (d), TAT-TY2 (e) and pristine TAT-H (f), Spiro-OMeTAD (g), TAT-TY1 (h) and TAT-TY2 (ı).

Table S1: Product cost analysis of TAT-TY1 and TAT-TY2 including reagents, solvents and intermediate products (Sigma Aldrich).

**Chemical Name**	**Reagent mass (g)**	**Solvent vol. (ml)**	**Reagent cost ($/g)**	**Solvent cost ($/ml)**	**Regagent + solvent cost ($)**	**Product cost ($/g)**
Oxindole	10		8.05		80.05	
POCl3	150		1.8		270	
NaOH	150		0.02		3	
Silica	125		0.07		8.75	
Methanol		250		0.02	5	
CH2Cl2		2500		0.02	50	
**Total**	**485**	**5000**			**416**	**109.4**
**Synthesis of TAT**	**(Yield=40% . 3.8 g)**					
Sodium Hydride	0.071		0.18		0.01	
Anhydrous DMF		10		0.19	1.9	
1-bromohexane	0.660		0.14		0.09	
Anhydrous MgSO4	0.5		0.10		0.05	
Silica	125		0.07		8.75	
Hexane		400		0.09	36	
Dichloromethane		100		0.02	2	
Total	**8.23**	**510**			**48.8**	**48.8**
**TAT-H**	**(Yield=75. 1g)**					
NBS	0.426		0.12		0.05	
Chloroform		40		0.09	3.6	
Dichloromethane		90		0.02	1.8	
Dimethylformamide		5		0.19	0.95	
Anhydrous MgSO4	0.5		0.1		0.05	
Silica	10		0.07		0.7	
Total	**11**	**135**			**7.15**	**5.9**
**TAT-HBr**	**(Yield= 80%. 1.2g)**					
Carbazole	2.5		0.2		0.5	
Sodium Hydride	1		0.071		0.071	
1-bromohexane	3		0.660		1.98	
DMF		30		0.19	5.7	
Chloroform		45		0.09	4.05	
Anhydrous MgSO4	0.5		0.1		0.05	
**Total**	7	75			**12.35**	**4.1**

**n-hexylcarbazole**	**(Yield=80% . 3 g)**					
N-bromosuccinimid	2.1		0.426		0.89	
Chloroform		45		0.09	4.05	
Diethyleter		45		0.05	2.25	
Anhydrous MgSO4	0.5		0.1		0.05	
**Total**	**2.6**	**90**			**7.24**	**2.89**
**3-bromo-N-hexylcarbazole**	**(Yield= 75%. 2.5 g)**					
n-BuLi (2.5 M)	2.1		0.19		0.39	
Triisopropyl borate	3		0.29		0.87	
Tetrahydrofuran		30		0.19	5.7	
Dichloromethane		145		0.02	2.9	
Hexane		200		0.09	18	
**Total**	**5.1**	**375**			**27.86**	**18.57**
**N-hexylcarbazole-3-ylboronic acid**	**(Yield= 60%. 1.5 g)**					
9-(4-Bromophenyl)carbazole	1		18.4		18.4	
n-BuLi (2.5M)	1.3		0.19		0.24	
Triisopropyl borate	1.8		0.29		0.15	
THF		18		0.19	3.42	
DCM		90		0.02	1.8	
Hexane		125		0.09	11.25	
**Total**	**4.1**	**233**			**35.26**	**22.03**
**(4-(9H-carbazol-9-yl)phenyl)boronic acid**	**(Yield= 63%. 1.6g)**					
$Pd(PPh_3)_4$	0.080		22.72		1.81	
K2CO3	0.5		0.08		0.04	
Toluene		15		0.08	1.2	
MgSO4	0.5		0.1		0.05	
Hexane		28		0.09	2.52	
Chloroform		115		0.09	10.35	
**Total**	**1.08**	**158**			**15.97**	**80**
**TAT-TY2 (HTM)**	**(Yield=66% . 0.198 g)**					
**Cost of TAT-TY1**						**273.12**
**Cost of TAT-TY2**						**269.6**

Table S2: Summary of undoped device parameters of HTMs.

Molecules	Scan direction	J_{SC} (mA/cm^2)	V_{oc} (mV)	FF (%)	PCE
**Spiro-OMeTAD**	Forward	1.65	1040	0.59	1,02
**TAT-H**	Forward	9.27	140	0.36	0.95
**TAT-TY1**	Forward	1.75	1040	0.15	0.28
**TAT-TY2**	Forward	0.98	810	0.25	0.20

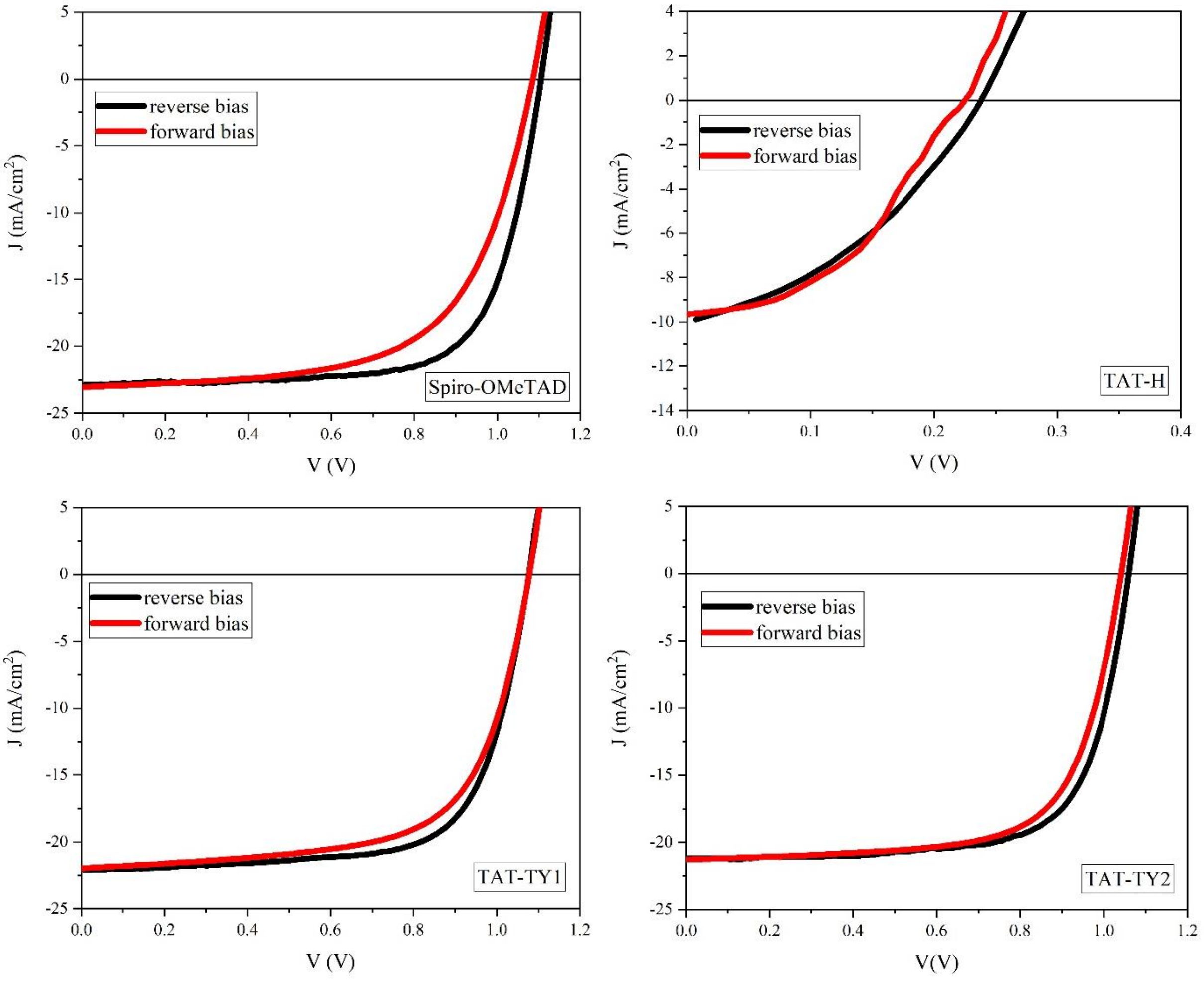

Figure S38. Forward and reverse bias J-V characteristics of PSCs prepared with a) Spiro-OMeTAD, b) TAT-H, c) TAT-TY1 and d) TAT-TY2.

Supplementary References:

(1) Rakstys, K.; Abate, A.; Dar, M. I.; Gao, P.; Jankauskas, V.; Jacopin, G.; Kamarauskas, E.; Kazim, S.; Ahmad, S.; Grätzel, M.; Nazeeruddin, M. K. Triazatruxene-Based Hole Transporting Materials for Highly Efficient Perovskite Solar Cells. *J. Am. Chem. Soc.* **2015**, *137* (51), 16172–16178. https://doi.org/10.1021/jacs.5b11076.

(2) Connell, A.; Wang, Z.; Lin, Y. H.; Greenwood, P. C.; Wiles, A. A.; Jones, E. W.; Furnell, L.; Anthony, R.; Kershaw, C. P.; Cooke, G.; Snaith, H. J.; Holliman, P. J. Low Cost Triazatruxene Hole

Transporting Material for >20% Efficiency Perovskite Solar Cells. *J. Mater. Chem. C* **2019**, *7* (18), 5235–5243. https://doi.org/10.1039/c8tc04231d.

(3) Illicachi, L. A.; Urieta-Mora, J.; Calbo, J.; Aragó, J.; Igci, C.; García-Benito, I.; Momblona, C.; Insuasty, B.; Ortiz, A.; Roldán-Carmona, C.; Molina-Ontoria, A.; Ortí, E.; Martín, N.; Nazeeruddin, M. K. Azatruxene-Based, Dumbbell-Shaped, Donor–π-Bridge–Donor Hole-Transporting Materials for Perovskite Solar Cells. *Chem. - A Eur. J.* **2020**, *26* (48), 11039–11047. https://doi.org/10.1002/chem.202002115.

(4) Kil, D. R.; Lu, C.; Ji, J. M.; Kim, C. H.; Kim, H. K. Dopant-Free Triazatruxene-Based Hole Transporting Materials with Three Different End-Capped Acceptor Units for Perovskite Solar Cells. *Nanomaterials* **2020**, *10* (5). https://doi.org/10.3390/nano10050936.

(5) Ramos, F. J.; Rakstys, K.; Kazim, S.; Grätzel, M.; Nazeeruddin, M. K.; Ahmad, S. Rational Design of Triazatruxene-Based Hole Conductors for Perovskite Solar Cells. *RSC Adv.* **2015**, *5* (66), 53426–53432. https://doi.org/10.1039/c5ra06876b.

(6) Tavasli, M.; Bettington, S.; Bryce, M. R.; Batsanov, A. S.; Monkman, A. P. Practical Syntheses of N-Hexylcarbazol-2-Yl- and -3-Yl-Boronic Acids, Their Cross-Coupled Products and a Derived Tris-Cyclometalated (Pyridin-2-Yl) Carbazole Iridium(III) Complex. *Synthesis (Stuttg).* **2005**, No. 10, 1619–1624. https://doi.org/10.1055/s-2005-865307.

(7) Ho, P. Y.; Wang, Y.; Yiu, S. C.; Kwok, Y. Y.; Siu, C. H.; Ho, C. L.; Lin Lee, L. T.; Chen, T. Photophysical Characteristics and Photosensitizing Abilities of Thieno[3,2-b]Thiophene-Based Photosensitizers for Photovoltaic and Photocatalytic Applications. *J. Photochem. Photobiol. A Chem.* **2021**, *406* (October 2020). https://doi.org/10.1016/j.jphotochem.2020.112979.

(8) Tavasli, M.; Moore, T. N.; Zheng, Y.; Bryce, M. R.; Fox, M. A.; Griffiths, G. C.; Jankus, V.; Al-Attar, H. A.; Monkman, A. P. Colour Tuning from Green to Red by Substituent Effects in Phosphorescent Tris-Cyclometalated Iridium(Iii) Complexes of Carbazole-Based Ligands: Synthetic, Photophysical, Computational and High Efficiency OLED Studies. *J. Mater. Chem.* **2012**, *22* (13), 6419–6428. https://doi.org/10.1039/c2jm15049b.

(9) Saliba, M.; Matsui, T.; Seo, J. Y.; Domanski, K.; Correa-Baena, J. P.; Nazeeruddin, M. K.; Zakeeruddin, S. M.; Tress, W.; Abate, A.; Hagfeldt, A.; Grätzel, M. Cesium-Containing Triple Cation Perovskite Solar Cells: Improved Stability, Reproducibility and High Efficiency. *Energy Environ. Sci.* **2016**, *9* (6), 1989–1997. https://doi.org/10.1039/c5ee03874j.

(10) Liu, D.; Li, S.; Zhang, P.; Wang, Y.; Zhang, R.; Sarvari, H.; Wang, F.; Wu, J.; Wang, Z.; Chen, Z. D. Efficient Planar Heterojunction Perovskite Solar Cells with Li-Doped Compact TiO2 Layer. *Nano Energy* **2017**, *31* (August 2016), 462–468. https://doi.org/10.1016/j.nanoen.2016.11.028.
